# Highly stable β-ketoenamine-based covalent organic frameworks (COFs): synthesis and optoelectrical applications

**DOI:** 10.1007/s12200-022-00032-5

**Published:** 2022-09-19

**Authors:** Yaqin Li, Maosong Liu, Jinjun Wu, Junbo Li, Xianglin Yu, Qichun Zhang

**Affiliations:** 1 Key Laboratory for Green Chemical Process of Ministry of Education, School of Chemical Engineering and Pharmacy, Wuhan Institute of Technology, Wuhan, 430074 China; 2 School of Chemistry and Environmental Engineering, Wuhan Institute of Technology, Wuhan, 430074 China; 3 Department of Materials Science and Engineering, City University of Hongkong, Hong Kong SAR, 999077 China; 4 Center of Super-Diamond and Advanced Films (COSDAF), City University of Hongkong, Hong Kong SAR, 999077 China

**Keywords:** Covalent organic frameworks, β-ketoenamine, Sensors, Energy storage, Batteries, Photocatalysis

## Abstract

Covalent organic frameworks (COFs) are one class of porous materials with permanent porosity and regular channels, and have a covalent bond structure. Due to their interesting characteristics, COFs have exhibited diverse potential applications in many fields. However, some applications require the frameworks to possess high structural stability, excellent crystallinity, and suitable pore size. COFs based on β-ketoenamine and imines are prepared through the irreversible enol-to-keto tautomerization. These materials have high crystallinity and exhibit high stability in boiling water, with strong resistance to acids and bases, resulting in various possible applications. In this review, we first summarize the preparation methods for COFs based on β-ketoenamine, in the form of powders, films and foams. Then, the effects of different synthetic methods on the crystallinity and pore structure of COFs based on β-ketoenamine are analyzed and compared. The relationship between structures and different applications including fluorescence sensors, energy storage, photocatalysis, electrocatalysis, batteries and proton conduction are carefully summarized. Finally, the potential applications, large-scale industrial preparation and challenges in the future are presented.

**Graphical Abstract:**

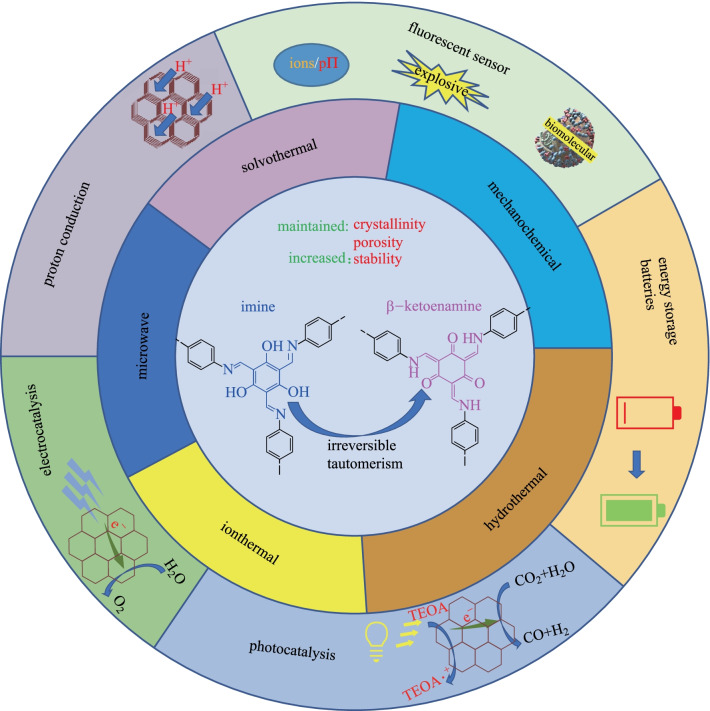

## Introduction

Covalent organic frameworks (COFs) are a new type of organic porous material, constructed through covalent bonds and various designed monomers [1–5]. Benefitting from their superior advantages such as outstanding stability, tunable pore size and structure, high crystallinity, regular internal channels, high surface area and low density [6–11], COFs have gained increasing attention and enormous progress in many fields including energy storage [12, 13], batteries [14, 15], adsorption [16, 17], separation [18, 19], sensing [20–22], drug delivery [23–25], and catalysis [26, 27], etc.

The majority of COFs applications correspond to the relevant positions of groups in the internal structure, thus it’s possible for researchers to design special monomers according to the desired performance [28, 29]. However, choosing functional groups, researchers have to consider different reaction types of the system [30]; if linkages are formed by irreversible reactions involving covalent bonds that would tend to form polymeric structures with a short-range structural order [31–33]. The growth of COFs depends on the reversible covalent bond formation reaction because high crystallinity strongly relays on structure repairing and assembly in a reversible condensation reaction, such as borate condensation reactions [34], Schiff-base condensation reactions [35], and Knoevenagel condensation reactions [36]. Besides the reversible reactions, the matching of shapes and angles of the monomers should also be considered for the formation of covalent bonds [37]. So, it’s still a huge challenge to select suitable monomers and appropriate reactions to form thermodynamically stable crystalline architectures.

Depending on the different type of reactions, the resulting linkages also vary. The first COF connected by boroxine and boronate ester rings was synthesized by the Yaghi group in 2005 [38]. Boron-based COFs are susceptible to hydrolyzation under basic or acidic conditions and sometimes even by moisture in air although they are thermally stable and highly crystalline [39, 40]. Triazine-based COFs are another type of COFs with good solvent stability, thermal stability and large conjugate system, but exhibit poor crystalline performance [41]. Schiff-base chemistry has also been used for the construction of imine-based COFs [42]. Except for the above-mentioned linkages, other types such as hydrazone linkage [43], azine linkage [44], imide linkage [45], squaraine linkage [46], olefin linkage [47], cyanovinylene linkage [48] and heterocycle-based linkage [49], have also been reported, where these types of linkages could be formed in one step. Moreover, post-synthesis modification is another effective method to change the type of linkage, such as: (1) The imine bond can be converted into amide linkages by oxidation [50]; (2) The C=N bond can be changed into a C–N bond by sodium borohydride reduction, and the as-formed COFs can be constructed through a single bond [51]; (3) The COFs containing imine bond or acetylene group can be transformed into quinoline-linked COFs by D-A cycloaddition reaction [52]; (4) The imine bond can also be converted into thiazole, imidazole, or oxazole [53–56]. These benzoheterocycles retain the crystallinity and porosity of the original COFs, while increasing the stability and π electron delocalization effect of COFs.

Imine-bond based COFs are the most reported COFs. As far as we know, according to the structural design, scientists have developed a variety of aldehyde or amino monomers for the synthesis of imine-based COFs, and these exhibit a variety of physical and chemical properties and potential applications [57]. In addition, imine-bonded COFs can be constructed in different environments, and the resulted COFs can exist in the form of powders [58], nanosheets [59], films [60] and foams [61]. Nevertheless, the stability of imine-based COFs is challenged when operating under harsh conditions such as the presence of strong acids, bases, or redox agents [62]. At the same time, imine-linked COFs are less thermally stable than their boronate-ester-linked COFs [63]. Additionally, 2D imine COFs exhibit the limited in-plane π-conjugation due to the inherent polarization of the C=N connection [64].

**Fig. 1:**
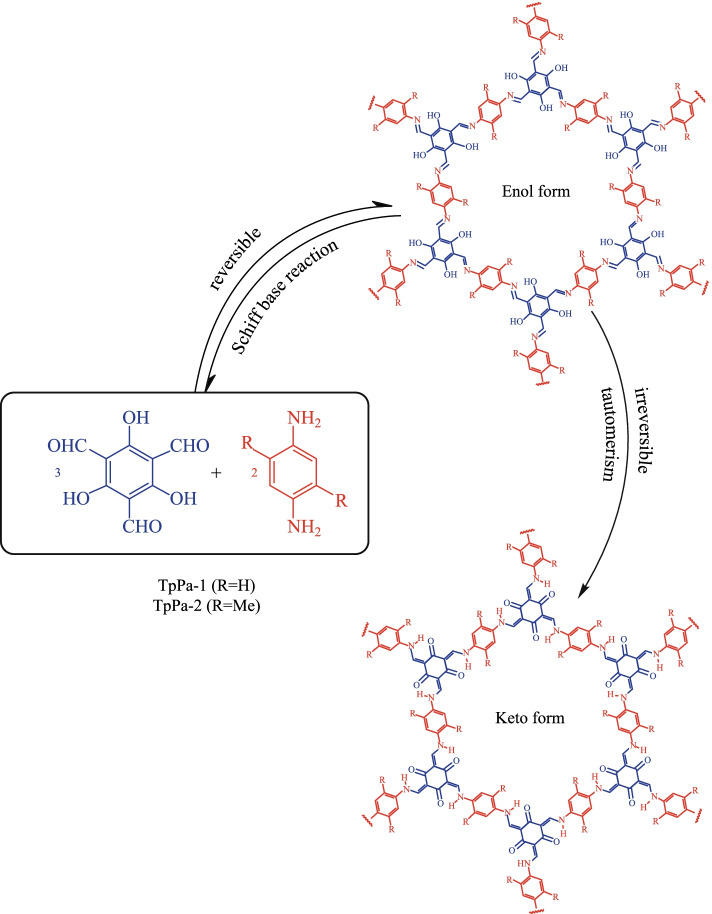
Schematic representation of the synthesis of **TpPa-1** and **TpPa-2** by the combined reversible and irreversible reaction. Reprinted with permission from Ref. [70]. Copyright 2012, American Chemical Society

Thus, many strategies have been adopted to enhance the stability of imine COFs [65]. Among them, the β-ketoenamine-based COFs are among the most stable of the reported imine-based COFs [66]. Unlike other imine-based COFs, the formation of this kind of COFs could be divided into two steps [67]: the first step is a reversible process, which induces the formation of a crystalline framework by the classical reversible Schiff-base reaction. Then, an irreversible process from the enol-imine (OH) form to keto-enamine (NH) form is conducted without loss of crystallinity. This kind of irreversible formation of keto-enamine (NH) structure enhances the stability of COFs, including stability in boiling water, even in strong acid or strong base solution. Hitherto, instability of COFs has been one of the key barriers to their application [68]. Actually, in 2003, the MacLachlan group firstly found the complete formation of NH salicylideneanilines (keto-enamine (NH) form) when 1,3,5-triformylphloroglucinol (**TP**) condensed with aniline derivatives [69]. Nearly ten years later, the Banerjee group (in 2012) [70] successfully introduced this keto-enamine (NH) form into the COF skeleton by using the reaction between **TP** and p-phenylenediamine (**PA-1**) or 2,5-dimethy-p-phenylenediamine (**PA-2**) (Fig. 1). As expected, only the keto-enamine form was observed in COF skeletons (**TpPa-1** and **TpPa-2**). With **TP** as basic monomer, different imine-bond-containing crystalline COFs were obtained, where the enol-imine (OH) would be irreversibly transformed into keto-enamine (NH) form without destruction of crystallinity. Those kinds of COFs were called as β-ketoenamine-based COFs. Since these β-ketoenamine-based COFs exhibit high stability, many of them have been constructed and the synthetic methods and the possible applications have been fully investigated. In this review, the related synthetic methods and applications for β-ketoenamine based COFs are summarized. Furthermore, the potential applications and challenges in the future have also been discussed.

## Synthetic method for preparation of β-ketoenamine-based COFs

At present, the most commonly used synthetic method for preparing β-ketoenamine-based COFs is the solvothermal method. Other methods such as mechanical grinding, microwave-assisted synthesis, ionthermal synthesis, hydrothermal synthesis, and interface synthesis, were also investigated in this study.

### Preparation of powder

#### Traditional solvothermal synthesis

The general operation of solvothermal method preparing for β-ketoenamine-based COF is as follows [71]: (1) adding the organic monomer TP, organic amine monomer, and catalyst into a Pyrex tube containing non-aqueous solvent; (2) dispersing the solution by ultrasonication thoroughly; (3) freezing the solution, in the glass tube, in liquid nitrogen, then vacuumizing, filling the tube with inert gas three times and thawing; (4) setting the reaction temperature and reaction time; (5) separating and purifying the as-prepared COF by filtration, centrifugation, solvent washing, and Soxhlet extraction.

**Fig. 2:**
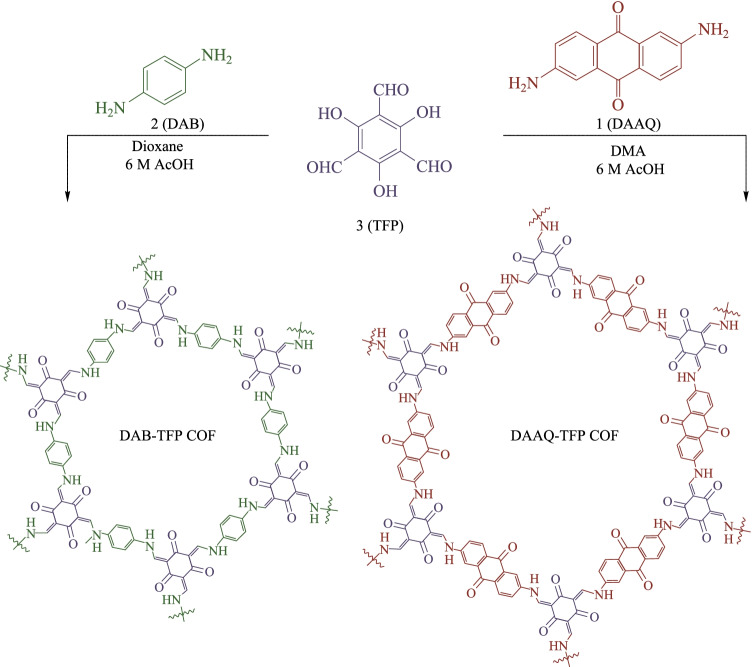
Synthesis of **DAB-** and **DAAQ-TFP COF**. Reprinted with permission from Ref. [74]. Copyright 2013, American Chemical Society

The solvothermal reaction takes place in a sealed and negative pressure environment, which is conducive to the full reversible reaction, long reaction time and sufficient crystallization time [72]. The β-ketoenamine-based COFs could be obtained with high crystallinity and relatively high porosity. In addition, the type and proportion of solvents also affect the crystallinity of the product The common solvent is the mixture of 1,4-dioxane and mesitylene, the catalyst is acetic acid in aqueous solution, the reaction temperature is 120 °C, and the reaction time is about 72 h. The first β-ketoenamine-based COFs (**TpPa-1** and **TpPa-2**) [70] were obtained by using the solvothermal method through a reversible condensation reaction between **TP** and **PA-1** or **PA-2** in the mixed solvent of mesitylene and dioxane (*v*:*v* = 1:1). The Brunauer–Emmett–Teller (BET) surface areas of the as-prepared COFs were found to be 535 and 339 m^2^/g for **TpPa-1** and **TpPa-2**, respectively. **TpPa-1** and **TpPa-2** showed strong resistance to boiling water and acid (9 N HCl). Moreover, **TpPa-2** showed higher stability than **TpPa-1** in base (9 N NaOH) solution. Zhou et al. [73] obtained the COF **PPN-31** by the solvothermal method with a suspension of trans-1,4-cyclohexanediamine and **TP** in a 60:1 (*v*:*v*) mixture of N,N-dimethylformamide (DMF) and 6 M^1^ aqueous acetic acid to give a crystalline red powder in 82% yield. Dichtel et al. [74] prepared the **DAAQ-TFP COF** and **DAB-TFP COF** by condensing either 2,6-diaminoanthraquinone (**DAAQ**) or p-diaminobenzene (**DAB**) with **TP** under a 20:1(*v*:*v*) mixture of dioxane (DMF for **DAAQ-TFP COF**) and 6 M AcOH; the yields of **DAAQ-TFP COF** and **DAB-TFP COF** were 70% and 85%, respectively. The structures of these two COFs are shown in Fig. 2. In addition, the BET surface areas of **DAB-TFP COF** and **DAAQ-TFP COF** were 365 and 1800 m^2^/g and pore size distribution of **DAAQ-TFP COF** was 20 Å. Because of the high specific surface area and large pore size, **DAAQ-TFP COF** had more exposed redox active groups than **DAB-TFP COF**, resulting in the higher capacitance of **DAAQ-TFP COF**.

Yan et al. [75] solvothermally synthesized BF-COFs from the suspension of 1,3,5,7-tetraaminoadamantane (**TAA**) and 1,3,5-triformylbenzene (**TFB**) or **TP** in a 10:1 (*v*:*v*) mixture of mesitylene and 3 M aqueous acetic acid. The BET surfaces were found to be 730 m^2^/g for **BF-COF-1** and 680 m^2^/g for **BF-COF-2**. The total pore volumes were evaluated to be *V*_p_ = 0.43 cm^3^/g for **BF-COF-1** and 0.39 cm^3^/g for **BF-COF-2** (*P*/*P*_0_ = 0.90). Both **BF-COFs** showed a narrow pore width (8.3 Å for **BF-COF-1** and 8.1 Å for **BF-COF-2**), which was in agreement with the pore size predicted from the crystal structures (7.8 Å for **BF-COF-1** and 7.7 Å for **BF-COF-2**).

**Fig. 3:**
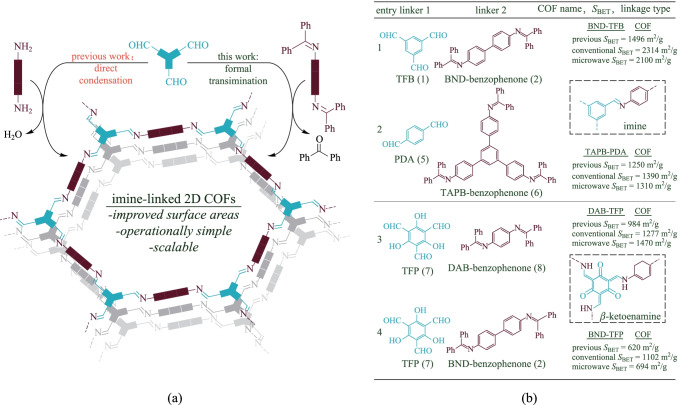
**a** Comparison of the synthesis of imine-linked 2D COFs from polyfunctional aryl amine monomers and the corresponding benzophenone imines. **b** COF scope evaluation under conventional heating and microwave irradiation. Reprinted with permission from Ref. [77]. Copyright 2017, American Chemical Society

The amine is a key factor to construct different β-ketoenamine-based COFs with different functions. The limited availability and poor oxidative stability of amines hamper the design and synthesis of the imine-linked COFs. Compared to free amines, the N-aryl benzophenone imines are more stable [76]. Thus, Vitaku and Dichtel [77] constructed the imine and β-ketoenamine-based COFs based on the stable monomer N-aryl benzophenone imines (Fig. 3a), the imine- and β-ketoenamine-based COFs obtained with this strategy showed higher crystal quality and this method was valid using both solvothermal and microwave methods (Fig. 3b).

**Fig. 4:**
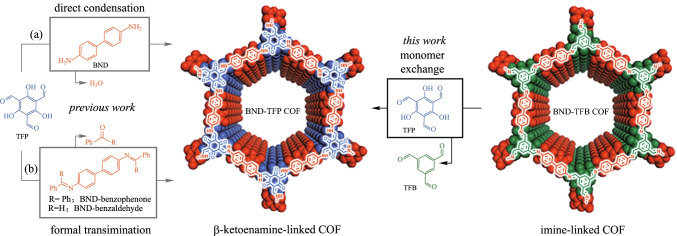
Preparation of β-ketoenamine-based 2D COFs from direct condensation, formal transformation, and monomer exchange approaches. Reprinted with permission from Ref. [78]. Copyright 2019, The Royal Society of Chemistry

The transformation from enol-imine (OH) form to keto-enamine (NH) form is irreversible, which may reduce the crystal quality in β-Ketoenamine-based COFs to some extent. Dichtel et al. [78] reported a strategy to improve the crystal quality for β-ketoenamine-based COFs in Fig. 4, where the condensation of **TFB** with benzidine (**BND)** was conducted to obtain a high crystalline quality **BND-TFB COF** that was subsequently exchanged with **TP** to form β-ketoenamine-based COFs (**BND-TFP COF**) with any changes in the crystalline quality.

**Fig. 5:**
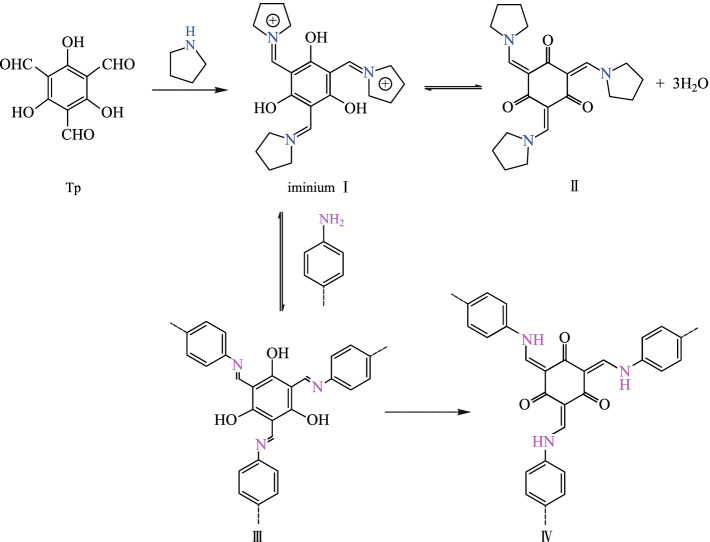
Preparation of β-ketoenamine-based 2D COFs using pyrolidine. Reprinted with permission from Ref. [79]. Copyright 2021, The Royal Society of Chemistry

Guo et al. [79] successfully prepared **TPBD-COF** between the **TP** and **BND** in a mixture of pyrrolidine (Py), n-BuOH and dichlorobenzene (1/1/9, *v*:*v*). The replacement of acetic acid aqueous solution with pyrrolidine plays an important role in the synthesis to allow the improvement of structural ordering and intrinsic porosity (Fig. 5). The calculated BET surface areas of **TPBD-COF** reached as high as 2157 m^2^/g, which was roughly four times higher than that achieved by using HOAc as a catalyst (572 m^2^/g). Moreover, the yield of **TPBD-COF** (89%) is higher than that by using HOAc as a catalyst (85%).

**Fig. 6:**
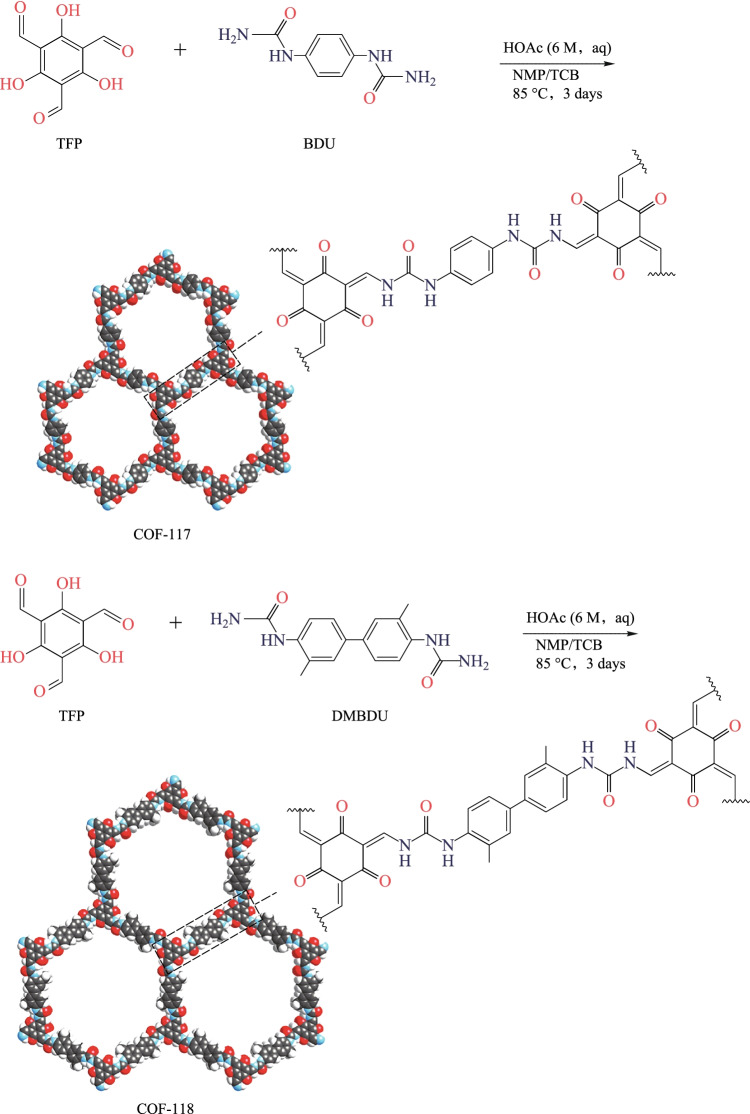
Synthesis of Urea-Linked COFs. Reprinted with permission from Ref. [80]. Copyright 2018, American Chemical Society

Yaghi et al. [80] synthesized urea-linked COFs (**COF-117** and **COF-118**) for the first time in 2018 through the Schiff-base condensation reaction of **TP** and urea-functionalized monomer (1,4-phenylenediurea for **COF-117**; 1,1′-(3,3′-dimethyl-[1,1′-biphenyl]-4,4′-diyl) diurea for **COF-118**). By heating to 85 °C in a mixture of N-methyl-2-pyrrolidinone, 1,2,4-trichlorobenzene and 6 M aqueous acetic acid (8:2:1, *v*:*v*:*v*) for three days, two crystalline COFs with 72% yield for **COF-117** and 56% yield for **COF-118** were obtained and their structures are shown in Fig. 6. The crystalline **COF-117** gradually became amorphous upon desolvation, and the Brunauer–Emmett–Teller (BET) surface areas (*S*_BET_s) of activated **COF-117** and **COF-118** samples were found to be 114 and 1524 m^2^/g respectively. The remarkable difference of crystallinity and surface area between the activated **COF-118** and **COF-117** can be partly attributed to the interlayer stabilization effect of the biphenylene linker used in the **COF-118**, which limits the shrinkage process during activation. **COF-117** is more inclined to undergo structural deformation caused by hydrogen bonding due to its higher weight percentage of urea groups. Moreover, **COF-117** is more likely to undergo structural deformation, resulting from the stronger hydrogen bond interaction among the urea groups in **COF-117**.

Although solvothermal synthesis is the most commonly used method to prepare this kind of COF, it does possess several disadvantages including harsh reaction conditions, complex reaction operation, and long reaction time. These disadvantages limit the large-scale synthesis and industrialization of this kind of COF. Therefore, researchers tried to explore other synthetic methods beyond solvothermal synthesis.

#### Mechanochemical synthesis

**Fig. 7:**
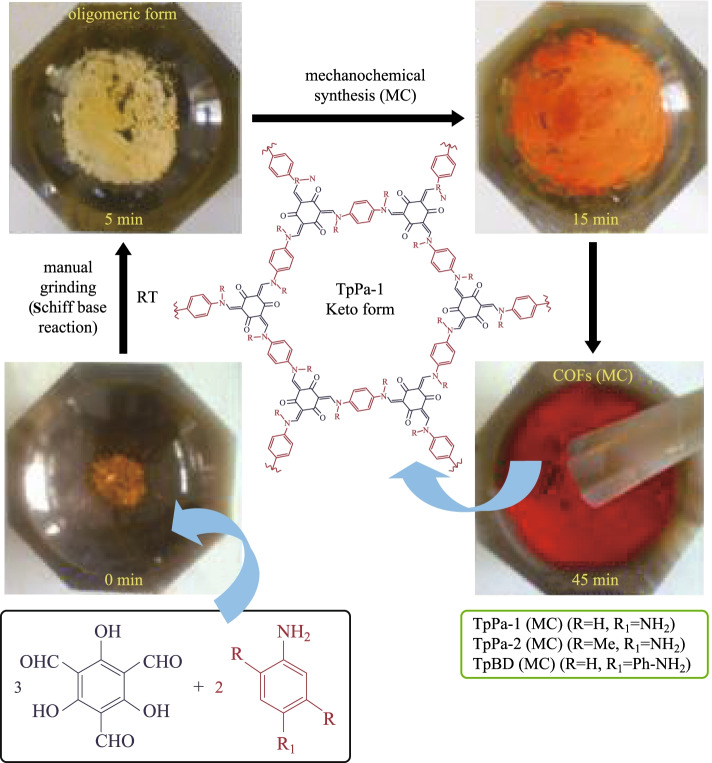
Schematic representation of the MC synthesis of **TpPa-1(MC)**, **TpPa-2 (MC)**, and **TpBD (MC)**. Reprinted with permission from Ref. [81]. Copyright 2013, American Chemical Society

Actually, mechanochemical synthesis has been widely used to construct porous polymers since it possesses the advantages of easy operation, mild reaction condition and low energy consumption. Banerjee et al. [81] first tried to introduce a simple, solvent-free, room-temperature mechanochemical (MC) synthetic method for preparing COFs. **TP** and **Pa-1** (for **TpPa-1**), **PA-2** (for **TpPa-2**), and **BD** (for **TpBD**) were placed in a mortar and grounded at room temperature to obtain the resulting COFs and the yield of the three COFs was close to 90%. The grinding process and color changes are shown in Fig. 7. However, the COFs synthesized by a mechanochemical method obviously exhibit relative lower BET surface areas (61, 56, and 35 m^2^/g for **TpPa-1 (MC)**, **TpPa-2 (MC)**, and **TpBD (MC)**, respectively) than the previously reported COF synthesized by solvothermal method. This may be due to the long-range pores in the MC, such that COFs were hindered by exfoliated COFs or oligomeric impurities in the MC process. Besides, powder X-ray diffraction (PXRD) was also performed, where the first peak of MC synthetic has relatively low intensity, which could be due to the random displacement of the 2D layers. It should be pointed out that the crystallinity and porosity of these mechanochemically synthesized COFs are unsatisfactory. Moreover, the authors found that some nanobelts can also be formed by this process, whereas they could use the mechanical method to exfoliate several β-ketoenamine-based COFs powder successfully [82].

**Fig. 8:**
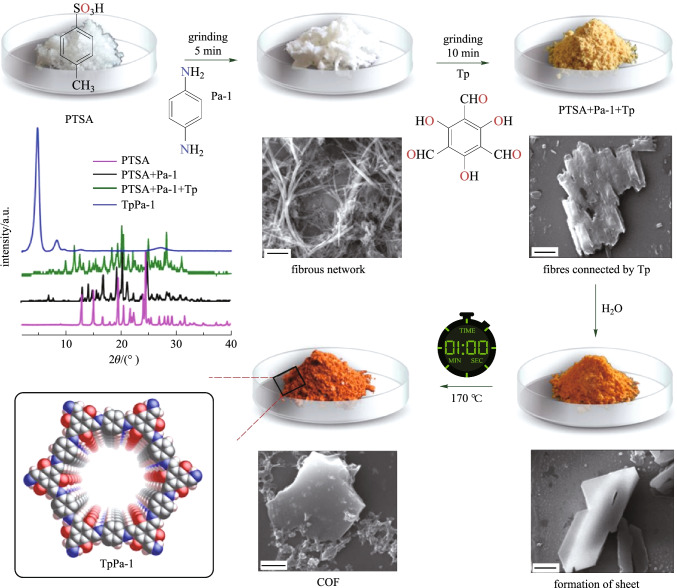
Synthesis of COFs via a molecular organization approach with sequential SEM and PXRDs of each individual crystallization steps. Reprinted with permission from Ref. [88]. Copyright 2017, American Chemical Society

Qin et al. [83] also successfully synthesized **COF-TpMA** by the mechanochemical (MC) grinding between the **TP** and melamine (**MA**). The as-prepared **COF-TpMA (MC)** shows small BET surface area (85 m^2^/g), with pore diameter of 0.64 nm, and the total pore volume of 0.35 cm^3^/g at *P*/*P*_0_ = 0.99. The crystallinity could be enhanced with longer grinding time (within 12 h), however, excessive grinding time will degrade the crystallinity and the material will eventually form its amorphous phase. In 2014, Banerjee et al. [84] reported the synthesis of 2D-COFs through a liquid-assisted grinding (LAG) method via adding a catalytic amount of organic solvents that allowed more reactant molecules to interact with each other easily during grinding. The as-obtained COFs exhibited higher crystallinity, higher purity and better yield compared to those prepared by the bare MC method. Nevertheless, these COFs (made from LAG) still showed low crystallinity and smaller porosity compared to the solvothermally synthesized COFs although the reaction time had been greatly reduced. In addition to the above characteristics, another important feature is that mechanical grinding could also be used to prepare sulfonated COFs, which is difficult to obtain by the solvothermal method [85]. Zhao et al. [86] successfully synthesized **NUS-9** and **NUS-10** by means of the condensation reaction of **TP** with 2,5-diaminobenzenesulfonic acid (**DABA**) or 2,5-diaminobenzene1,4-disulfonic acid (**DABDA**) with a small amount of mixed solvents, and using mechano-assisted synthesis. Such sulfonated COFs are difficult to prepare under solvothermal conditions (Fig. 8). The BET surface areas of as-prepared COFs were 102 and 69 m^2^/g for **NUS-9** and for **NUS-10**, respectively. In general, poor crystallinity and the porosity are the common disadvantages of the mechano-assisted synthesis. However, such poor porosity may also be an advantage in other properties of the material. Banerjee et al. [87] synthesized a bipyridine-functionalized COF via mechanochemical routes (**TpBpy-MC**) by mixing **TP** and 2,2'-bipyridine-5,5′-diamine (**BPY**) through the classic Schiff-base reaction. The **TpBpy-MC** is a good proton-conducting solid electrolyte candidate due to its poor porosity and compacting pellet, which might inhibit the fuel crossover; this is opposite to the properties of **TpBpy-ST** synthesized by solvothermal method with good porosity. This also proves that mechanical synthesis of COF is an important complementary method to the solvothermal technique. Banerjee et al. [88] also developed one method of the grinding the mixture first and then heating it to construct COFs with high BET surface areas. They first ground p-toluene sulphonic acid and corresponding diamine thoroughly with a pestle, then **TP** was added into the mixture followed by the addition of a little amount of water (~ 100 μL). The reaction mixture was thoroughly ground, and was then heated at 170 °C for 60 s. The as-prepared powder was dipped into hot water to isolate porous COFs containing high crystalline structure with more than 90% yield. Using this method, more than 12 highly crystalline COFs with surface areas as high as 3109 m^2^/g were obtained. The BET surface area increased almost 2–3 times compared to their previously-reported solvothermal counterparts.

#### Microwave synthesis

Wei et al. [89] synthesized **TpPa-COF** under microwave (MW) heating conditions between **Pa-1** and **Tp** in a mixture of mesitylene:1,4-dioxane: 3 M acetic acid = 3:3:1 (*v*:*v*:*v*). By microwave irradiation at 100 °C for 60 min, a red powder was obtained with 83% yield, which is much higher than the yield (8%) of **TpPa-COF (CE)** obtained by heating with an oil bath in a sealed tube at 100 °C for 60 min. The BET surface area of **TpPa-COF (MW)** (724.6 m^2^/g) was higher than **TpPa-COF** (prepared by traditional solvothermal method, 535 m^2^/g) and **TpPa-COF (CE)** (162.6 m^2^/g). Xu et al. [90] synthesized **TpPa-2 (MW)** in an open microwave system at 100 °C for 60 min with microwave power of 500 W, based on the condensation of **TP** and **Pa2** in the presence of a mixed solvent (2.5 mL) with mesitylene:dioxane:3 M AcOH = 3:3:1 (*v*:*v*:*v*). The *S*_BTE_ of **TpPa-2 (MW)** was found to be 535.2 m^2^/g, which was higher than the reported **TpPa-2(MC)**, **TpPa-2(ST)** and the synthesized **TpPa-2(MC)**. Moreover, **TpPa-2(MC)** was successfully synthesized by microwave irradiation within 60 min, and the reaction rate was ~ 72 times faster than that for the reported **TpPa-2(ST),** prepared by solvothermal method with a time of 72 h, which probably contributed due to the high nucleation or crystal growth rate under microwave irradiation.

#### Ionthermal synthesis

**Fig. 9:**
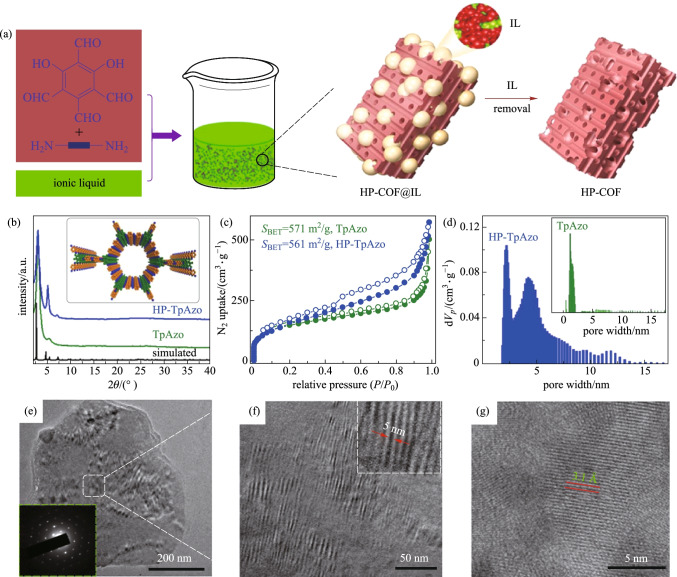
**a** Synthesis of **HP-TpAzo**. **b** PXRD patterns of **HP-TpAzo** and **TpAzo**. **c** N_2_ sorption isotherms of **HP-TpAzo** and **TpAzo** at 77 K. **d** DFT pore size distribution of **HP-TpAzo** and **TpAzo**. **e**–**g** High resolution transmission electron microscopy (HRTEM) images of **HP-TpAzo**. Reprinted with permission from Ref. [92]. Copyright 2020, The Royal Society of Chemistry

Ionthermal synthesis method is a green and facile method for preparing COFs. Dong et al. [91] reported the condensation of **TP** and **PA-1**, **PA-2**,4,4'-azodianiline (**AZO**) and 1,5-diamino-4,8-dihydroxyanthraquinone (**AHAn**) by using ionic liquid such as 3-methylimidazolium hydrogen sulfate [BSMIm]HSO_4_ as a solvent to prepare **TFP-PA-COF**, **TFP-MPA-COF**, **TFP-Azo-COF** and **TFP-AHAn-COF** with yields of 86%, 79%, 84%, and 73%, respectively. The BET surface areas were calculated to be 446, 604, 809, and 363 m^2^/g for **TFP-PA-COF**, **TFP-MPA-COF**, **TFP-Azo-COF**, and **TFP-AHAn-COF**, respectively. These values were comparable to those for COFs with the same structures but prepared under solvothermal condition (the reported BET surface areas were 535, 339, and 1328 m^2^/g for **TFP-PA-COF**, **TFP-MPA-COF**, and **TFP-Azo-COF**, respectively). Moreover, ionic liquids could be recycled for further use without the loss of activity. Wang et al. [92] developed a simple, and environmentally-friendly method for the synthesis of **HP-TpAzo** through the condensation reaction of **TP** and **AZO** under mild conditions in ILs [C_*n*_mim][BF_4_] (*n* = 4, 6, 10; “*n*” is the alkyl chain length of the ILs) without the need of additional template at 50 °C. Figure 9 shows the synthetic route and the characterization of **HP-TpAzo**. The yield of **HP-TpAzo** was 92%, and the BET surface area was found to be 561 m^2^/g, which is similar to that of **HP-TpAzo** prepared by the solvothermal method (571 m^2^/g). Notably, the **HP-COFs** prepared in ionic liquids exhibit large mesopores and maintain the original microporosity time because ionic liquids could also act as templates beyond as solvents and catalysts during the reaction process. In addition to the original structure-dominated micropores in COFs, the properties of the pores can be tuned by the alkyl chain length of ionic liquids, resulting in even larger mesopores.

Zhao et al. [93] also chosed an IL ([BMIm][HSO_4_]) as a solvent to synthesize **TFP-EB** and **TFP-DAAQ** via the condensation reaction of **TP** and ethidium bromide (**EB**) or **DAAQ**, where all reaction mixtures required to be degassed through freezing-pump-thaw cycles, sealed, and heated at 120 °C for three days. The as-obtained samples showed mixed pores with poor surface areas (12.5 and 22.4 m^2^/g for **TFP-EB** and **TFP-DAAQ**, respectively). The PXRD patterns exhibited a broad (001) diffraction peak, indicating a serious aggregation phenomenon.

#### Hydrothermal synthesis

**Fig. 10:**
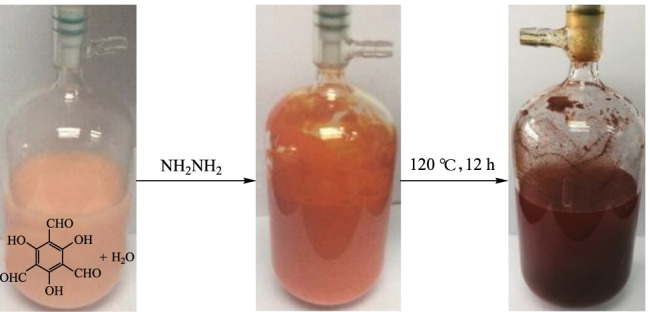
Photographs showing the progress of a 10-g-scale synthesis of **HCOF-1**. Reprinted with permission from Ref. [95]. Copyright 2019, The Royal Society of Chemistry

Several COFs, such as **TpPa-1**, **TpPa-2**, **TpBD**, **TpFn**, **DAAQ** and **TpBpy**, were prepared by Banerjee et al. [94] in hydrothermal conditions. The as-prepared COFs showed high crystallinity although the surface area and porosity were similar to those of their solvothermal counterparts. The hydrothermal method provides a green and large-scale-synthesis possibility for preparing COFs in water without the use of organic solvents. Xu et al. [95] reported the synthesis of **HCOF-1** by the condensation of **TP** and hydrazine hydrate with water as the solvent. The **HCOF-1** possessed higher crystallinity and larger specific surface area than the counterparts obtained in organic solvents. Moreover, the production efficiency is also promoted (several days decreased to several hours) under the hydrothermal method. More importantly, hydrothermal synthesis breaks through the limitation of solvothermal synthesis, and could provide a green and feasible method to prepare COF in large-scale (Fig. 10).

### Fabrication of films

#### *In situ* growth

**Fig. 11:**
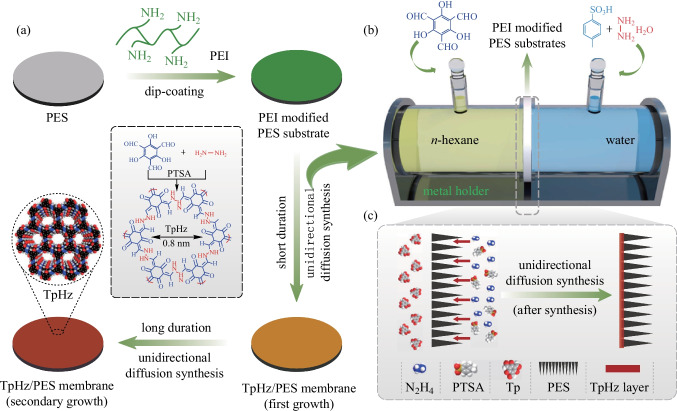
**a** Formation of **TpHz/PES** membrane on the PEI-modified PES substrate. **b** Diffusion cell for **TpHz** growth on the substrate by unidirectional diffusion synthesis. **c** Formation of the **TpHz** selective layer on the substrate top side. Reprinted with permission from Ref. [97]. Copyright 2020, Elsevier

In addition to the preparation of powder COF, more and more attention has been paid to the fabrication of COF film. Dichtel et al. [96] prepared **DAAQ-TFP** films, which were formed through the slow introduction of **TP** monomer into a DMF solution of **DAAQ** in the presence of Au substrates (90 °C for 3 h). Varying the initial monomer concentration provided control of the thickness of the resulting films. The films grown at an initial **DAAQ** concentration of 11 nM were 380 nm while films prepared at the concentration of 22 mM were 525 (484 ± 80) nm thickness. Thus, the adjustable film thickness and high specific surface area give this type of COF films high capacity. By using the unidirectional diffusion synthesis, Wang et al. [97] grew the **TpHz** selective layer on macroporous polymer substrates to form composite membranes for desalination. As shown in Fig. 11b, **TP** was fully dispersed in n-hexane, while hydrazine hydrate and p-toluene sulfonic acid (**PTSA**) were dissolved in DI water. The PEI-modified PES substrate was vertically fixed on a diffusion cell, and the setup was kept at room temperature to allow unidirectional diffusion synthesis three times for the formation of **TpHz/PES** membrane. Benefiting from the unidirectional diffusion synthesis, the single side growth of **TpHz** on the top side of the substrate was achieved (Fig. 11c). As a result, the as-formed **TpHz** selective layers were defect-free and very thin, enabling the fast permeation of water with the tight rejection to ions.

**Fig. 12:**
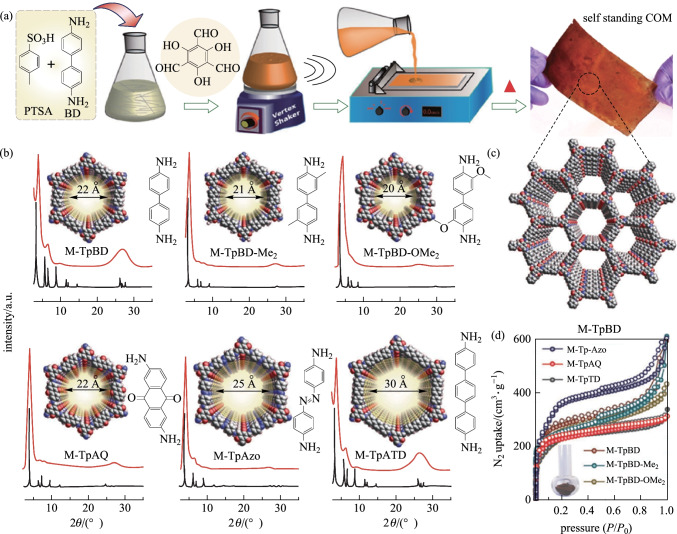
**a** COMs (**M-TpBD**) fabrication. **b** Comparison of the experimental and simulated PXRD. **c** Space-filling packing model of **M-TpBD** hexagonal framework. **d** Comparison of N_2_-adsorption isotherms of all six COMs. Reprinted with permission from Ref. [99]. Copyright 2017, WILEY–VCH Verlag GmbH & Co. KGaA

Pan et al. [98] solvothermally synthesized **TpHZ** red powder through the reaction between **TP** and hydrazine hydrate in a mixed solvent containing mesitylene/dioxane/6 M AcOH (5:5:1, v:v:v), followed by grinding for 45 min to obtain COF (**TpHZ**) nanosheets. Then, the COF nanosheet suspension and Alg solution were spin-coated on a hydrolyzed polyacrylonitrile (HPAN) membrane, followed by immersion it in CaCl_2_ solution to give the Alg-Ca/COF/HPAN membrane. The membrane thickness was less than 100 nm. Hydrogen bonding makes COF and Alg attach firmly to the surface of then HPAN membrane. The as-fabricated Alg-Ca layer provided a superhydrophilic surface, which could induce the porous COF layer to act as selective water channels. In 2017, Banerjee et al. [99] showed a new fabrication methodology to construct a series of self-standing, porous and crystalline COFs. Diamine and **TP** were added into the mixed solvent of **PTSA** and water, respectively. After mixed evenly, the as-obtained mixture was poured onto a glass plate and baked in an oven at 60–120 °C for 12–72 h to obtain the resultant COMs (Fig. 12), which displayed higher porosity and crystallinity over the reported powder form. These self-standing COMs are flexible, continuous, and devoid of any internal defects or cracks, and they show long-term durability and recyclability.

#### Interfacial polymerization

**Fig. 13:**
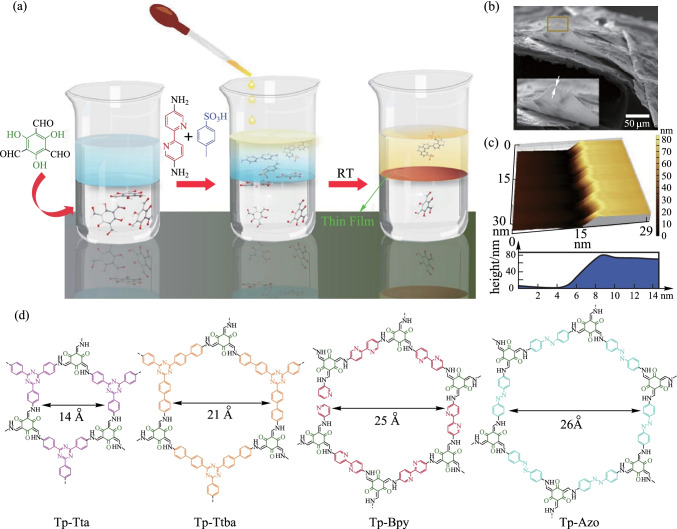
**a** Interfacial crystallization process for preparing **Tp-Bpy** **thin film**. **b** and **c** SEM and AFM images. **d** Structures of all the COFs. Reprinted with permission from Ref. [101]. Copyright 2017, American Chemical Society

Banerjee et al. [100] presented two different types of COF nanofilms (∼300 nm) constructed from the same building blocks. COF_fiber_ film was synthesized by interface synthesis, where **TP** dissolved in dichloromethane (DCM) and **AZO** or 3,8-Diamino-6-phenylphenanthridine (**DPP**) and **PTSA** were dissolved in water. The system was kept at room temperature for 72 h in undisturbed condition to obtain the **Tp-Azo**_**fiber**_ and **Tp-DPP**_**fiber**_ films. COF_sphere_ film was synthesized by the condensation reaction between the **TP** and diamine (**Azo** and **DPP**) in the dry DCM to reflux at 70 °C for 36 h (with trifluoroacetic acid as the catalyst). The BET surface areas calculated for the four thin films were 1556 (**Tp-Azo**_**sphere**_), 764 (**Tp-Azo**_**fiber**_), 805 (**Tp-DPPs**_**phere**_), and 489 m^2^/g (**Tp-DPP**_**fiber**_) with total pore volumes of 0.746, 0.563, 0.726, and 0.572 cm^3^/g, respectively. In the following year, his team [101] reported four COF thin films prepared using interfacial synthesis. As shown in Fig. 13a, TP was dissolved in DCM, **PTSA; BPY** or **AZO** were dissolved in water, or triamine [4,4',4″- (1,3,5-triazine-2,4,6-triyl) tris (1,1'-biphenyl) trianiline (**Ttba**); 4,4',4″-(1,3,5- triazine-2,4,6-triyl) trianiline (**Tta**)] was dissolved in water and acetonitrile. The reaction vessels were kept at room-temperature for 72 h to obtain the resulting COFs. The yields of the as-obtained COFs are 23% for **Tp-Bpy**, 46% for **Tp-Azo**, 37% for **Tp-Ttba**, and 15% for **Tp-Tta**. The BET surface areas calculated for these four thin-films are 1151 (**Tp-Bpy**), 647 (**Tp-Azo**), 626 (**Tp-Ttba**) and 333 m^2^/g (**Tp-Tta**), with the total pore volumes of 0.918, 0.491, 0.447, and 0.365 cm^3^/g, respectively.

**Fig. 14:**
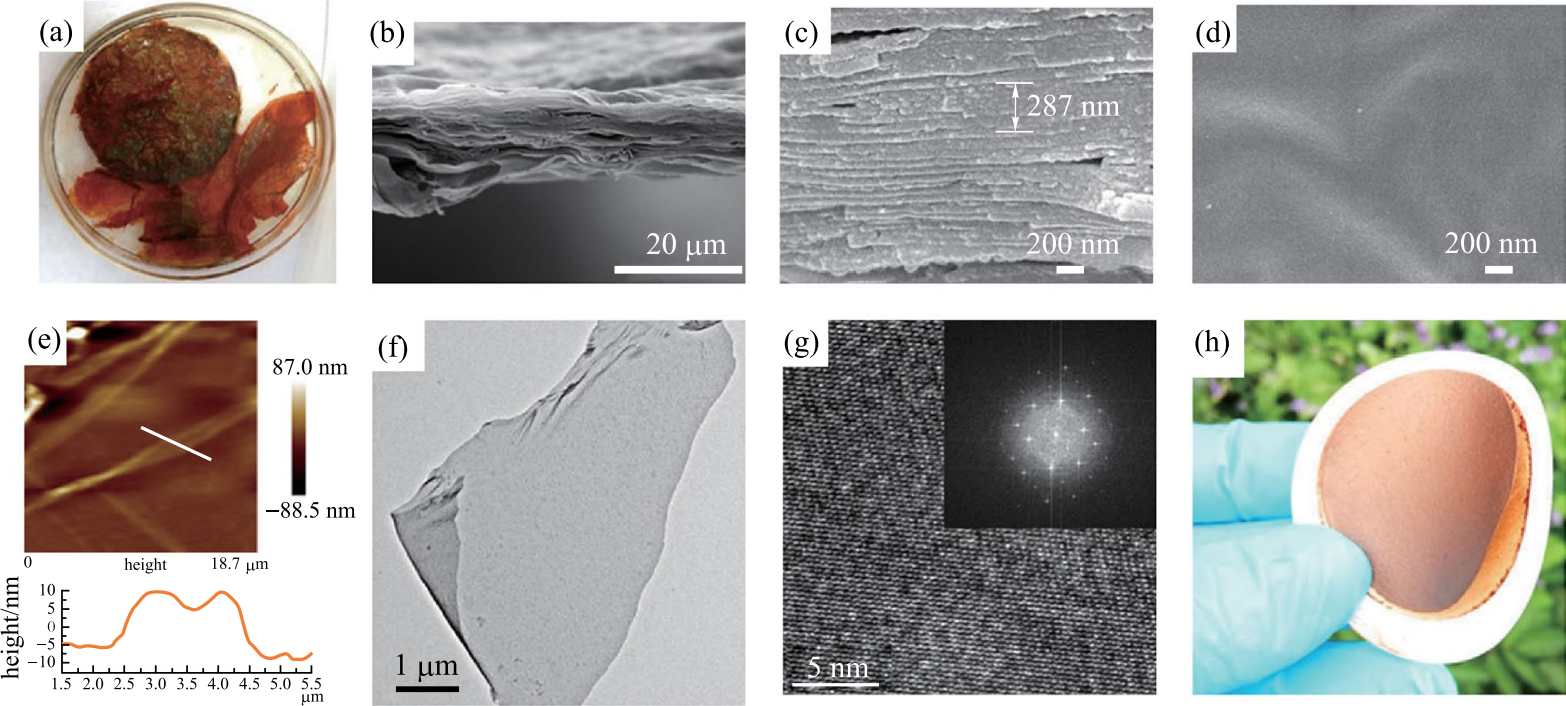
**a** Pristine **FS-COM-1**. **b** and **c** Cross-section SEM images of **FS-COM-1**. **d** Top-view SEM image of **FS-COM-1**. **e** AFM image. **f** TEM image. **g** HRTEM patterns. **h** Digital images of substrate-supported membrane (**FS-COM-1-VF**). Reprinted with permission from Ref. [102]. Copyright 2020, Springer Nature

Ma et al. [102] prepared **FS-COM-1** by a modified buffering interlayer interface (BII) method, where **TP** was dissolved in DCM. Then, 3–12 M acetic acid solution was added on the top of the DCM solution, followed by the drop-by-drop addition of tris (4-aminophenyl) amine (**TAPA**) in DMF onto the surface of acetic acid solution. After a period of time, the product was collected. Using this method, a buffering solution with a low-density solvent was chosen to separate these two miscible organic solvents to address the above-mentioned solubility problem. The BET surface area of **FS-COM-1** is 478 m^2^/g. **FS-COM-1** consists of tens of thinner nanosheets (Fig. 14), stacking on top of each other with a total thickness of 10–20 µm. The concentrations of acetic acid are important factors for the successful formation of COF membranes. For low acetic acid concentrations, only membranes were obtained (9 M gives a mixture of COF membrane and nanoparticles while 12 M provides only COF nanoparticles). When the concentration of aqueous acetic acid increased, the morphology of COF changed from membranes to nanoparticles. The possible reason is that the concentration of acetic acid affected the interface formation between acetic acid and dichloromethane, thus affecting the formation of the initial COF layer, eventually leading to the morphology changes of COFs from membranes to nanoparticles.

#### Mixed matrix membranes (MMMs)

Zhao et al. [103] synthesized **NUS-2** (**TP** and hydrazine hydrate) and **NUS-3** (**TP** and 2,5-diethoxy-terephthalohydrazide) by the solvothermal method. These COFs were then exfoliated into nanosheets and subsequently blended with poly (ether imide) (UItem) or polybenzimidazole (PBI). The mixed matrix membranes showed homogeneous textures, indicating the excellent compatibility between COF fillers and polymer matrixes. The surface areas were measured to be 415 and 757 m^2^/g for **NUS-2** and for **NUS-3**, respectively, which are comparable to other 2D COFs with hexagonal channels such as **DAAQ-TFP COF** (365 m^2^/g), **COF-LZU1** (410 m^2^/g), and **TpPa-1** (535 m^2^/g). The high *S*_BET_, good thermal stability (up to 300 °C) and excellent resistance toward hydrolysis in both neutral and acidic conditions endow MMMs with high stability and gas separation performance.

### COF foams

**Fig. 15:**
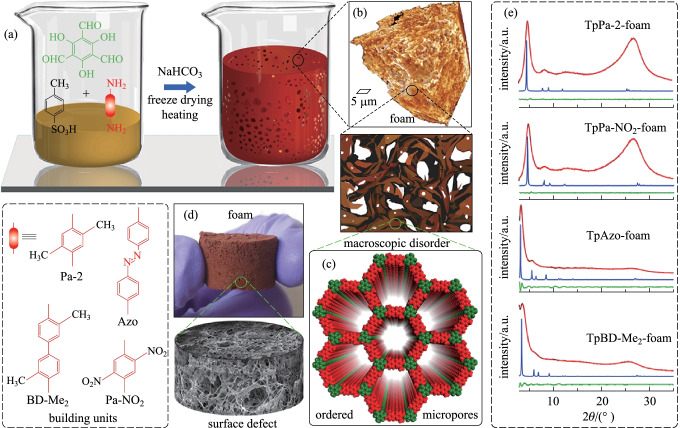
**a** Preparation of COF foam; **b** 3D volume rendered X-ray computed tomographic image of COF foam and cartoon representation; **c** Space-filled model of the COF foam; **d** Digital image and scanning electron microscopy (SEM) image of COF foam; **e** Powder X-ray diffraction (PXRD) pattern of the as-synthesized foams. Reprinted with permission from Ref. [105]. Copyright 2019, American Chemical Society

The extrinsic meso and microporosity of COF foam could produce a large surface area, which is beneficial for the maximum adsorption of guest molecules through various interactions [104]. Banerjee et al. [105] prepared four COF-foams (**TpPa-2-foam**, **TpPa-NO**_**2**_**-foam**, **TpAzo-foam**, **TpBD-Me**_**2**_**-foam**) according to an* in situ* gas-phase foaming protocol (Fig. 15a). **TpPa-2**, **TpPa-NO**_**2**_, **TpAzo**, and **TpBD-Me**_**2**_**-foams** show the surface areas of 579, 254, 1054, and 797 m^2^/g, respectively. Using a simple strategy involving the reaction of sodium bicarbonate (NaHCO_3_) with excess **PTSA** led to the continuous effervescence of CO_2_ to induce disorder within the ordered COF crystallites to provide tcrystalline COF-foams with hierarchical porosity.

**Fig. 16:**
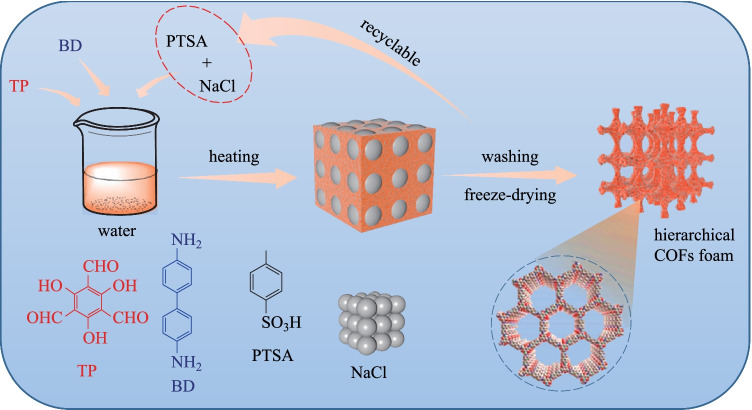
Synthesis of hierarchical porous COFs foams through NaCl template-assisted strategy. Reprinted with permission from Ref. [106]. Copyright 2021, Elsevier

Huang et al. [106] developed a template-assisted synthetic method for preparing HP-COF foams (**HP-TpBD**), where **PA-1** and **TP** were mixed in a mortar, NaCl was employed as hard template, and **PTSA** was utilized as a catalyst. Then, the mash was polymerized at 170 °C for 5 min. After washing and freeze-drying, **HP-TpBD-X** foams (X = 0, 300, 600, 900 mg, where X represents the amount of NaCl) were obtained (Fig. 16). The specific surface area (ranged from 679 to 743 m^2^/g) and pore size (almost all of them are 2.0 nm) confirmed that the long-range order channel of virgin **TpBD** is well maintained when micron-sized NaCl is added. The number of macropores increased with the increase of NaCl dosage. **HP-TpBD-0** composed abundant and regular nanoparticles (~ 50 nm), while **HP-TpBD-300** consisted of substantial nanofibers with microcrystals. The morphology of **HP-TpBD-600** changed from nanoparticles to nanofibers completely, which is similar behavior to that of **HP-TpBD-900**. Besides, the macropore volume of **HP-TpBD-900** was calculated to be 64.75%, which is much larger than that of the virgin **TpBD COF** (~ 11%) The macropores ranged in size from 50 to 250 μm. All these observations suggest that **HP-TpBD-900** possessed abundant and disordered hierarchical pores.

## Application

### Fluorescent sensor

#### Metal ion sensing

**Fig. 17:**
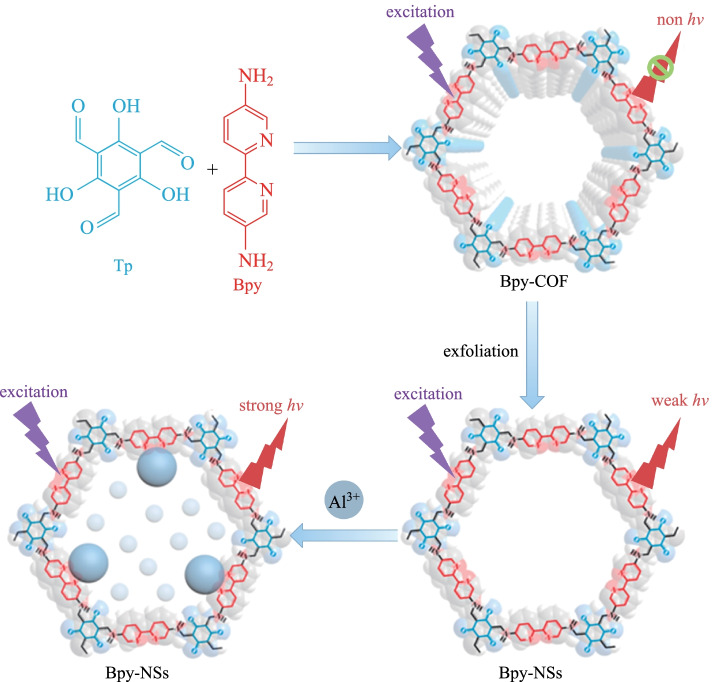
Preparation and Al^3^^+^ detection of **Bpy-NSs**. Reprinted with permission from Ref. [108]. Copyright 2019, American Chemical Society

The luminescence of two-dimensional COFs is easily quenched by interlayer π-π stacking or intramolecular rotation [107]. The as-prepared nanobelt can decrease the π-π stacking. Qiu et al. [108] employed **Bpy** and **TP** to synthesize **Bpy-COF** and the related nanobelt **Bpy-NS** was obtained by grinding and ultrasonic-assisted peeling (Fig. 17). The fluorescence intensity of **Bpy-NSs** increased linearly with the concentration of Al^3+^ and reached equilibrium at 350 μM. The fluorescence intensity increased by 15.7 times, and the fluorescence quantum yield increased to 1.74%.

**Fig. 18:**
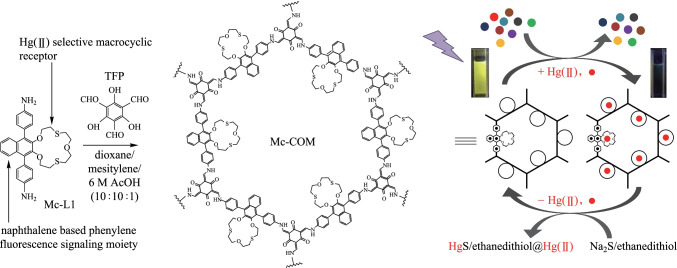
Structure of **Mc-CON** and removal of Hg^2^^+^ contamination from water. Reprinted with permission from Ref. [110]. Copyright 2021, American Chemical Society

Continuing on this direction, Qiu et al. [109] loaded AuNPs in situ onto **Tp-Bpy NSs**. The **AuNPs@Tp-Bpy** nanocomposite display high Hg^2+^ detection behavior, and the minimum detection limit is 0.33 nM. Due to the good affinity between Au and Hg, **AuNPs@Tp-Bpy** nanocomposites have excellent selectivity to Hg^2+^. The reusability of **AuNPs@Tp-Bpy** was evaluated, where the Hg^2 +^ regeneration experiments were carried out in water. After six cycles of repeated detection and mercury removal, the activity of **AuNPs@Tp-Bpy** still remained at 95.5%. Das et al. [110] reported a dithia-crown ether decorated β-Ketoenamine-based COF (**Mc-CON**), which could detect Hg^2+^ at the ppb level. The **Mc-CON** was synthesized by **TP** and naptho-dithia-crown ether-based macrocycle functionalized diamine (**Mc-L1**). The fluorescence intensity of the **Mc-CON** suspension gradually decreased with the continuous addition of Hg^2+^. The detection limit of **Mc-CON** for Hg^2+^ was calculated to be 45 ppb, which is equal to or better than many known thioether-substituted molecular sensors. The effect of other metal ions such as Ag^+^, Pb^2+^, Cd^2+^, Ca^2+^, Cu^2+^, Zn^2+^, Fe^2+^, Mn^2+^, K^+^ and Na^+^ on the detection of Hg^2+^ was negligible. As shown in Fig. 18, the detection mechanism may be the chelation of Hg^2+^ to the naphthalene-dithiocyanate crown ether receptor, which promotes the electron transfer from the extended β-ketoenamine-connected π-conjugated network containing electron-rich naphthyl triphenyl fluorescent units to the Hg^2+^ empty orbital, resulting in a large amount of fluorescence quenching.

#### Explosive sensing

In 2015, Murugavel et al. [111] reported two β-ketoenamine-based COFs (**TAPB-TFP** and **iPrTAPB-TFP**) as fluorescent sensors for polynitro compounds (PA, DNT, p-DNB and m-DNB). **TAPB-TFP** was a condensation product between 1,3,5-tris(4′-aminophenyl) benzene (**TAPB**) and **TP** under solvothermal conditions. While, **iPrTAPB-TFP** was synthesized from 1,3,5-tris(4′-amino-3′,5′-isopropylphenyl) benzene (**iPrTAPB**) and **TP** as polymerization monomers. In the presence of different concentrations of polynitro compounds (such as PA, DNT, p-DNB and m-DNB), the fluorescence was effectively quenched. It is worth noting that PA is the most effective quencher among all polynitroaromatic compounds, which may be due to the proton transfer from PA to the nitrogen atoms on the COF. Fluorescence test results showed that in the presence of 13 ppm of PA, the overall trend of the quenching order of COF with PA was **TAPB-TFP** > **iPrTAPB-TFP**. Compared with that of PA, the fluorescence quenching efficiency of other polynitroaromatic hydrocarbons was very low.

#### Biomolecule

**Fig. 19:**
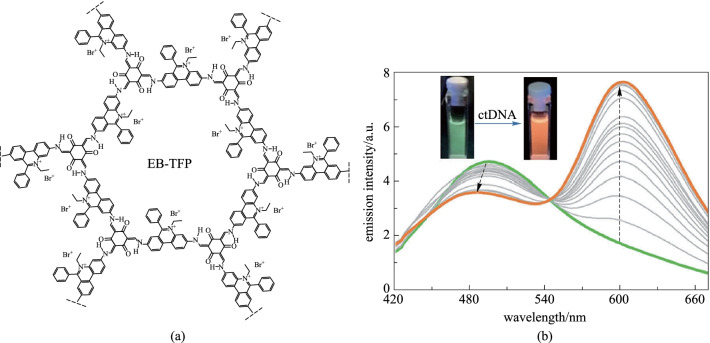
**a** Structure of **EB-TFP-ICON**; **b** Emission (*λ*_ex_ = 350 nm) spectral changes of **EB-TP-iCONs** upon addition of ctDNA (0–32 mm). Reprinted with permission from Ref. [112]. Copyright 2018, WILEY–VCH Verlag GmbH & Co. KGaA

Ajayaghosh et al. [112] prepared a covalent organic framework (**EB-TFP**) based on **EB** and **TP** (Fig. 19a). It can shed itself in water to produce two-dimensional ion covalent organic nanosheets (**EB-TFP-ICONs**) for selective detection of double-stranded DNA (dsDNA). In this case, there is a significant difference in the fluorescence intensity at 600 nm before and after the cDNA strand is added (Fig. 19b). For example, comparing the fluorescence intensity of **EB-TFP-ICON** in the presence of 20-mer ssDNA and its complementary strands, the fluorescence of the latter is nearly doubled. Detailed studies have shown that comparing with single-stranded DNA (ssDNA), the recombination phenomenon of dsDNA has a high degree of selectivity.

**Fig. 20:**
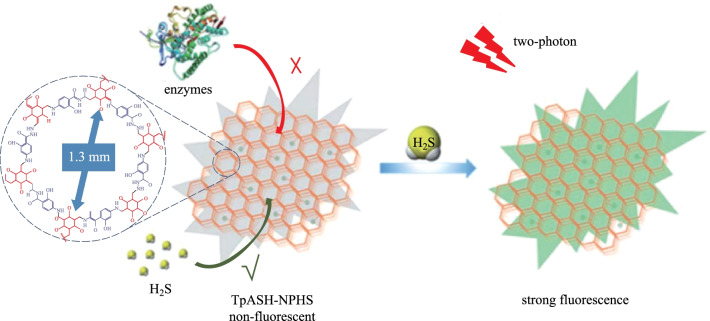
Schematic representation of two-photon fluorescent COF nanoprobes for the fluorescence sensing of H_2_S. Reprinted with permission from Ref. [113]. Copyright 2018, The Royal Society of Chemistry

H_2_S is a very important gas signal molecule in liver disease. Zhang et al. [113] reported a novel 2D COF (**TpASH-NPHS-COF**) nanoprobe condensation from 4-aminosalicylhydrazide (**ASH**) and **TP** via Schiff-base condensation. The bulk **TpASH-NPHS-COF** was then exfoliated to **TpASH-NPHS-COF** nanobelt via solvent-assisted exfoliation. **TpASH-NPHS** is harmless to the cell itself, and has excellent photostability and long-term biological imaging capabilities. More importantly, compared with small molecular probes, **TpASH-NPHS** is not interfered with by intracellular enzymes when detecting in cells. This allows **TpASH-NPHS** to be used to monitor the level of endogenous H_2_S in a mouse model of liver cirrhosis (Fig. 20). In the range of 0–25 μM, the fluorescence enhancement intensity of **TpASH-NPHS-COF** is directly proportional to the concentration of H_2_S. The lower detection limit of this substance is calculated to be 0.11 μM.

**Fig. 21:**
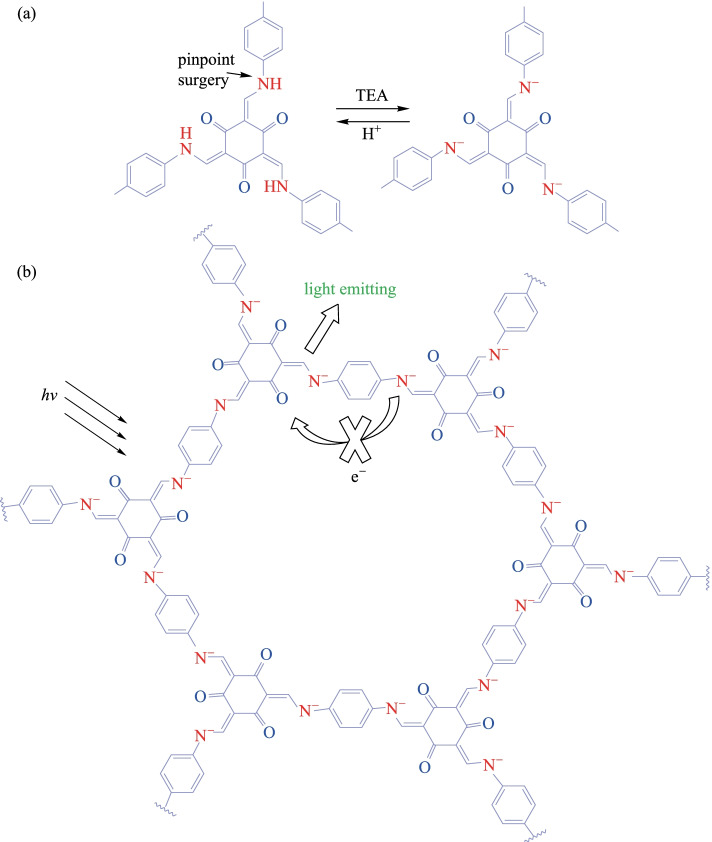
**a** N–H unit undergoes acid–base reaction; **b** mechanism for fluorescence enhancement of **TpPa-1** toward TEA. Reprinted with permission from Ref. [115]. Copyright 2019, American Chemical Society

In 2019, Yan et al. [114] developed a chemically stable Eu^3+^-modified COF named as **Eu@TpPa-1**. **Eu@TpPa-1** exhibits a turn-on fluorescence response to levofloxacin. It shows good sensitivity and rapid response to levofloxacin within 1 min, and at the same time, the interference of other coexisting species in serum and urine was inhibited. Its good selectivity and high anti-interference property make **Eu@TpPa-1** successful in detecting levofloxacin in serum and urine systems. Subsequently, Wang and Yan [115] found that **TpPa-1** could also be used to turn on the luminescence response to triethylamine (**TEA**) vapor, and its lower detection limit reached the ppm level (Fig. 21). In addition, the aqueous products of **TpPa-1** and **TEA** (recorded as **TpPa-1@LE**) could be further used for the quantitative tracking of the biomarker methylglyoxal (MGO) in the serum system. The minimum detection limit was 117.5 nM, and the detection range was 10^−6^–10^−2^ M, which meets the requirements for in vitro detection of MGO, showing great potential application in further diagnosis of diabetes.

#### pH detection

**Fig. 22:**
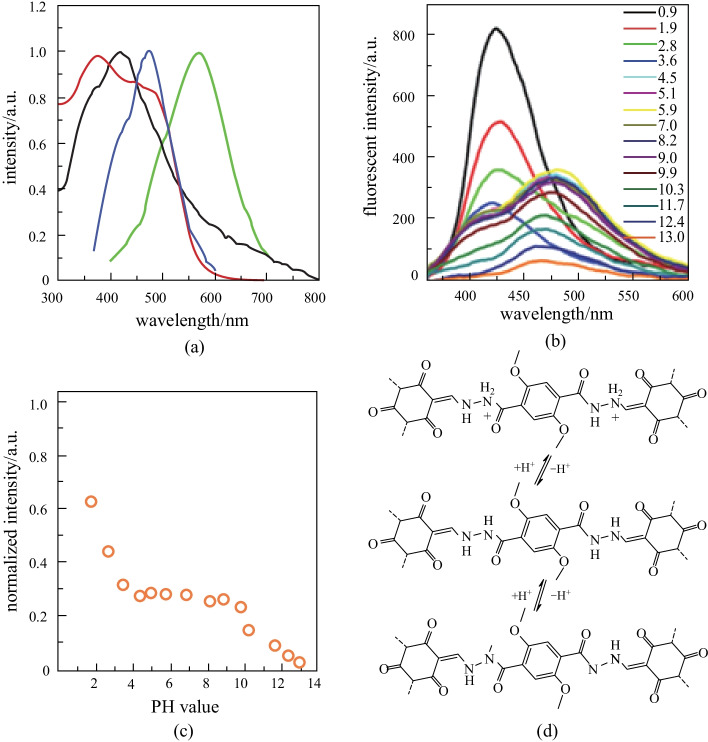
**a** Absorption (water: black line, solid state: red line) and fluorescence spectra (water: blue line, solid state: green line) of **COF-JLU4**; **b** pH dependent fluorescence of **COF-JLU4** in the aqueous solutions; **c** Photoluminescence intensity of **COF-JLU4** at 428 nm in the aqueous solutions with different pH values; **d** Deprotonation and protonation processes of the **COF-JLU4**. Reprinted with permission from Ref. [116]. Copyright 2016, Springer Nature

β-Ketoenamine-based COFs exhibit high stability in acidic solution, which might present the possibility of application as a pH detector. Liu et al. [116] used 2,5-dimethoxyterephthalohydrazide and **TP** as raw materials to synthesize **COF-JLU4** under solvothermal conditions. **COF-JLU4** has high crystallinity, good porosity and strong photoluminescence performance (Fig. 22a, b). Meanwhile, **COF-JLU4** exhibited excellent hydrolytic stability and dispersibility due to the ketoenamine form of the skeleton. The fluorescence intensity of **COF-JLU4** at different pH values was explored. The results indicate that the stronger the acidity, the stronger the fluorescence intensity of the solution. The solution with pH = 13.0 has the weakest fluorescence intensity (Fig. 22c). The protonation of nitrogen sites in acidic solutions can cause the blue shift and fluorescence enhancement of **COF-JLU4** (Fig. 22d).

Yin et al. [117] used **TP** and 9,9-dibutyl-2,7-diaminofluorene (**DDAF**) to synthesize **COF-4-OH** through the Schiff-base reaction. There are multiple active sites in **COF-4-OH**, such as imines, hydroxyl groups, and alkyl groups, which could allow it to be used for detection of a wide variety of other materials. Those researchers found that its intramolecular hydrogen bond generates a very good signal when detecting the water content in organic solvents. For example, in ethanol, as the water content increases, the fluorescence intensity at 400 nm will decrease and its minimum detection limit for water in ethanol is 0.03%. Secondly, **COF-4-OH** exhibits different fluorescence behaviors in different solvents, so **COF-4-OH** could be further used as a probe to distinguish different polar solvents. With the addition of water, the emission strength at 400 nm is enhanced in polar solvents, while the emission strength at 590 nm is decreased because the transfer of enol to ketone in polar solvents involves less proton transfer. Also, the presence of hydrogen bonds in **COF-4-OH** can allow it to be used to detect pH value. As the pH value increases, the emission strength at 400 nm is significantly enhanced while the emission at 590 nm remains stable, realizing the sensing of different pHs. This research group have proposed that the –OH group in the enol state forms hydrogen bonds with H^+^ to form −OH_2_^+^, leading to the reduced emission strength. As the pH value increases, −OH_2_^+^ returns to −OH, resulting in the improved emission strength.

### Energy storage

**Fig. 23:**
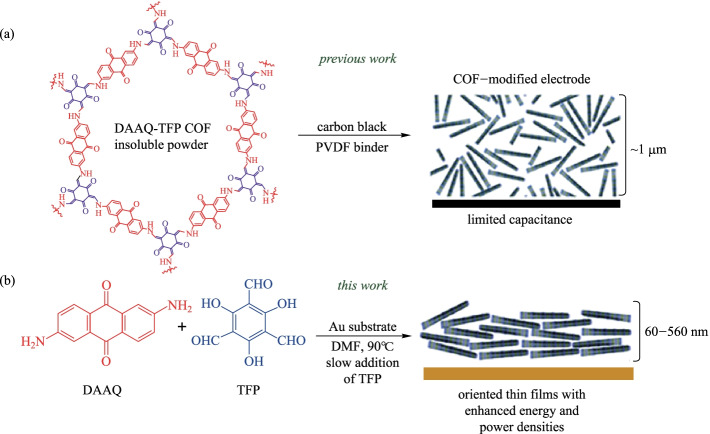
**a** Random orientation of **DAAQ–TFP COF** on the electrode; **b** An oriented thin film **DAAQ–TFP COF** on an electrode surface. Reprinted with permission from Ref. [96]. Copyright 2015, The Royal Society of Chemistry

Many redox groups can be introduced into the skeleton of β-ketoenamine-based COFs through the reaction between **TP** and various multifunctional diamines. At the same time, due to the unique irreversible tautomerism, these COFs can exhibit high stability in an acid or base environment. Dichtel et al. [74] integrated **DAAQ** (a redox active specie) into a β-ketoenamine-based 2D COFs and the as-obtained **DAAQ-TFP COF** displayed the initial specific capacitance of (48 ± 10) F/g which could be maintained after 5000 charge–discharge cycles. It is worth noting that the monomer **DAAQ** only exhibited a lower initial capacitance ((35 ± 7) F/g) and the specific capacitance further decreased quickly to (21 ± 3) F/g (which was just 60% of its initial specific capacitance) after several cycles. When another **DAB-TFP COF** without redox active part was used as an electrode in pseudocapacitor, its charge-storage performance was even worse (only (15 ± 6) F/g). Moreover, even in the H_2_SO_4_ electrolyte, all β-ketoenamine-based COFs provided stable specific capacitance for at least 5000 charge–discharge cycles. These results proved the application prospects of β-ketoenamine-based COFs in electrochemical energy storage devices. To increase the active centers in **DAAQ-TFP COF**, the Dichtel group [96] conducted the slow reaction of **TP** and **DAAQ** on the Au substrate to fabricate the **DAAQ-TFP COF film** (Fig. 23b). The as-obtained COF film could form more oxidation active sites than the bulky COF, and the specific capacitance was also an order of magnitude higher. The capacitance had nearly 400% increase when the electrodes were functionalized with orderly-oriented COF films compared to those functionalized with the randomly-oriented COF powder.

**Fig. 24:**
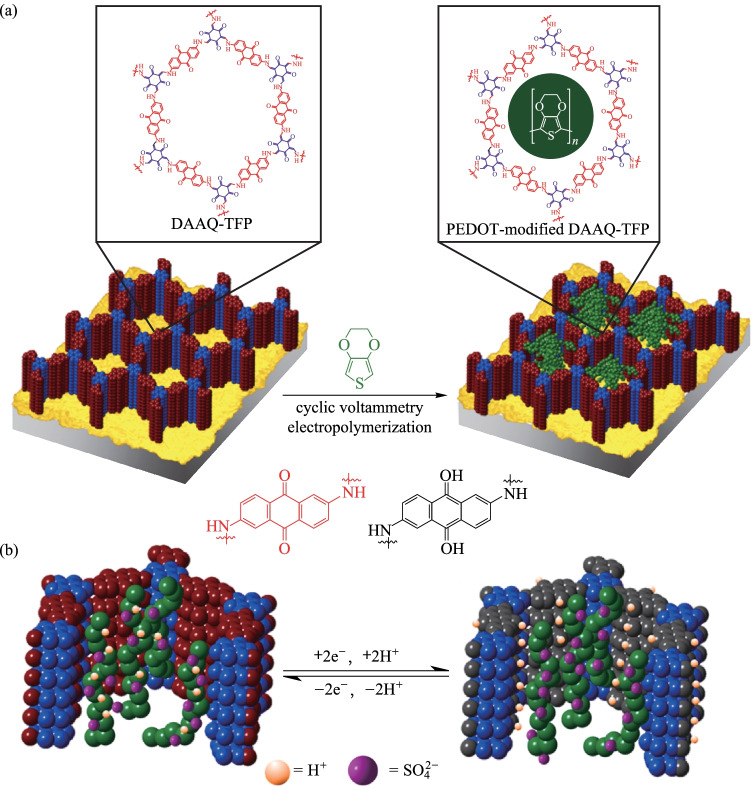
**a** Preparation of PEDOT-modified **DAAQ-TP COF** film by electropolymerization; **b** Cross section of the pore. Reprinted with permission from Ref. [118]. Copyright 2016, The Royal Society of Chemistry

In addition, integrating conductive polymers into the ordered channels of the COF framework could improve the conductivity of the redox COF film. Dichtel et al. [118] used the aforementioned **DAAQ-TFP** COF as a scaffold, doping poly(3,4-ethylene-dioxythiophene) (**PEDOT**) into the pores of the COF framework, and the modified COF shows strong electrochemical activity (Fig. 24). The electrochemical analysis showed that the capacitance (maximum capacitance was 350 F/cm^3^) after **PEDOT** modification was better than that of **DAAQ-TFP** COF film. Moreover, even 1 μm thick film could maintain a fast-charging rate without affecting the performance. Compared with the synthetic **DAAQ-TFP** COF film, this performance is equivalent to a 30-fold enhancement in volume energy density and a 12-fold increase in volume power density.

**Fig. 25:**
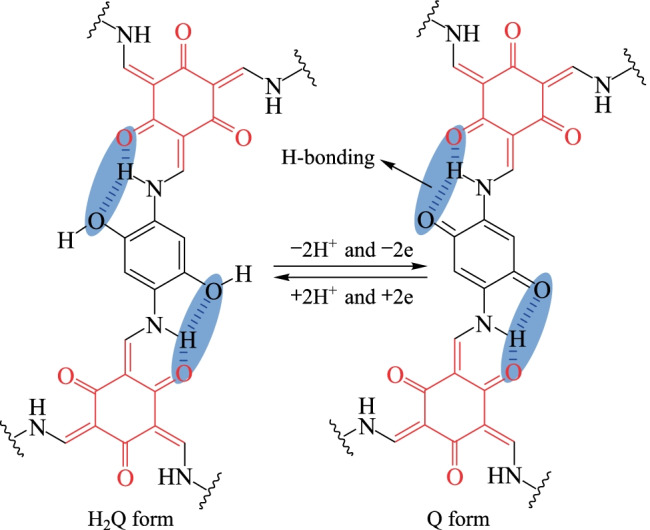
Proposed H-bonding stabilized both the hydroquinone (H_2_Q) and benzoquinone (Q). Reprinted with permission from Ref. [121]. Copyright 2017, American Chemical Society

However, the shortcomings of electropolymerization as well as the restricted requirement of COF film as a precursor made it very difficult to scale up the production and widen its applications. To solve these problems, in 2019, Awaga et al. [119] introduced conductive **PEDOT** into the order channels through in-situ solid-phase polymerization (SSP). The electrochemical properties of the as-obtained composite materials were analyzed. When the current density was 1 A/g, the calculated specific capacitance was 1663 F/g for **PEDOT@AQ-COF**, which was about sixfold higher than that of **AQ-COF/PEDOT** (274 F/g). When the current density increased to 500 A/g, the specific capacitance decreased to 998 F/g. This suggested that the electrode containing **PEDOT@AQ-COF** had ultra-fast charge and discharge capabilities. Some conductive polymers and carbon materials have been successfully functionalized with hydroquinone (H_2_Q) to improve pseudocapacitance [120]. However, most COFs electrode materials have insufficiently precise control over redox functionalization. Inserting the H_2_Q/Q electrochemical excitation process into the COF backbone, where the intramolecular H bond may provide better electrochemical stability with the system of H_2_Q/Q, can significantly increase the pseudocapacitance. Therefore, in 2017, Banerjee et al. [121] employed **TP** and 2,5-dihydroxy-1,4-phenylenediamine [**Pa-(OH)**_**2**_] as structural units to construct H_2_Q-based COF (**TpPa-(OH)**_**2**_**)** (Fig. 25). The capacitor performance of **TpPa-(OH)**_**2**_ showed that when the current density was 0.5 A/g, the maximum specific capacity of **TpPa-(OH)**_**2**_ is 416 F/g, the highest capacitance obtained by COF-based materials at that time. Subsequently, the specific capacitances of the non-functionalized **TpBD** and **TpBD-(OMe)**_**2**_ were 29 and 16 F/g, respectively, confirming that the redox conversion was the result of the action of phenol.

**Fig. 26:**
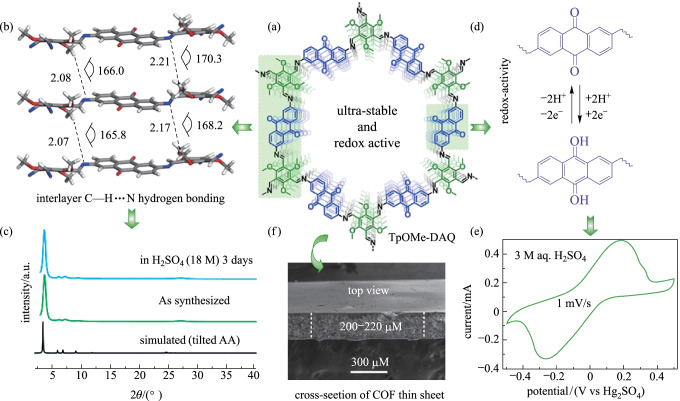
**a** Structure of **TpOMe-DAQ**; **b** structural illustration of the interlayer C–H…NH-bonding; **c** X-ray spectra of **TpOMe-DAQ**; **d** Description of charge–discharge; **e** Cyclic voltammetry of the as-synthesized sheet in 3 M H_2_SO_4_ using 1 mV/s^2^ potential scanning. Reprinted with permission from Ref. [122]. Copyright 2018, American Chemical Society

Poor electrochemical stability is a major problem in the application of COFs in supercapacitors. To address this issue, Banerjee et al. [122] reported a COF (**TpOMe-DAQ**) (Fig. 26), which combined redox active groups with hydrogen bonds. **TpOMe-DAQ** was synthesized through the reaction between 2,4,6-trimethoxy-1,3,5-benzenetricarbaldehyde (**TPOME**) and 2,6-diaminoanthraquinone (**DAQ**). The as-fabricated **TpOMe-DAQ** film as an electrode material in supercapacitors showed extremely high stability in concentrated acid. Specifically, when the concentration of electrolyte increased from 2 to 3 M, the capacitance increased from 13 to 169 F/g. Banerjee et al. attributed this increase in capacitance to the involvement of H^+^ ions in the redox active quinone (C=O) center.

**Fig. 27:**
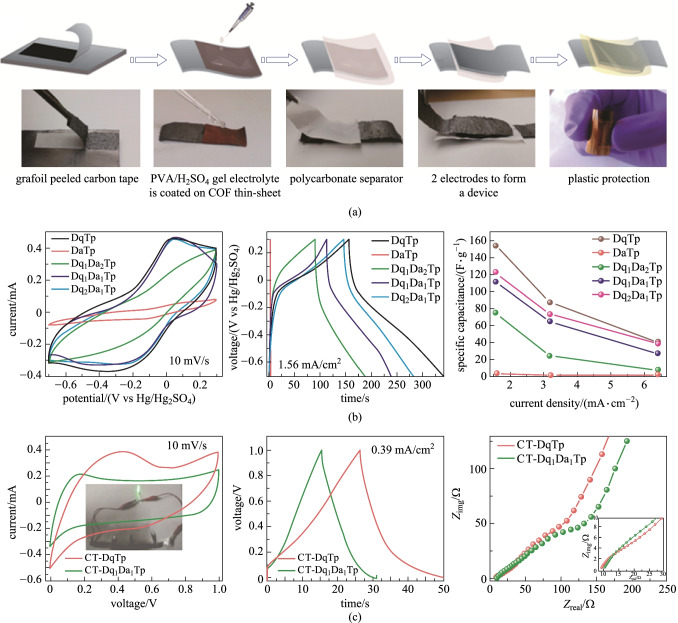
**a** Diagrammatic representation of the fabrication of the **CT**-**COF** supercapacitor device; **b** Three-electrode characterization using CV and CD; **c** Device characterization: *CV*, charge–discharge, and impedance analysis of **CT-DqTp** and **CT-Dq**_**1**_**Da**_**1**_**Tp** COF supercapacitor devices. Reprinted with permission from Ref. [126]. Copyright 2018, American Chemical Society

Flexible supercapacitors in modern electronic devices require high mechanical strength, flexibility, and independence [123–125]. However, it has been found that it is very difficult for one single electrode to have all the above-mentioned performance. Thus, a solid-state molecular baking strategy was used to synthesize stretchable and flexible COFs to address these challenges. In 2018, Banerjee et al. [126] reported that **DAQ** and 2,6-diaminoanthracene (**DA**) linkers were inserted into COFs through a solid-state molecular mixing process (Fig. 27a). The capacitance value of each COF chip was evaluated. Among them, due to the higher loading amount of anthraquinone linkers, **Dq**_**2**_**Da**_**1**_**Tp** showed better electrochemical performance. When the current density was 1.56 mA/cm^2^, the capacitance reached 122 F/g. Even the **Dq**_**1**_**Da**_**1**_**Tp** COF film with good mechanical properties also showed good specific capacitance.

### Photocatalysis

#### Photocatalytic hydrogen generation

**Fig. 28:**
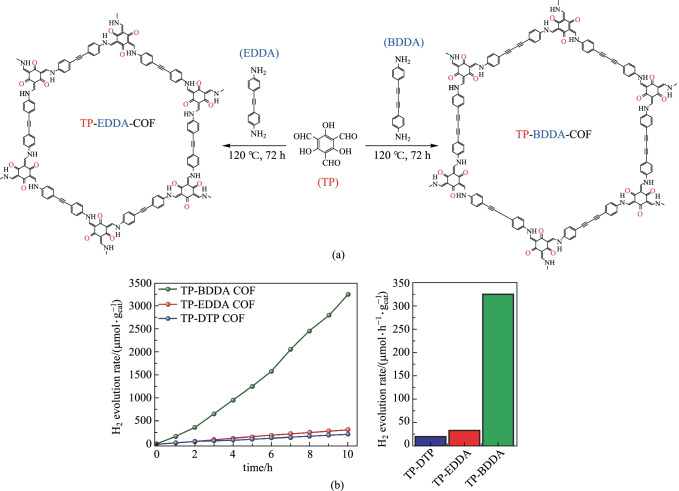
**a** Preparation of **TP-EDDA** and **TP-BDDA** COFs; **b** Comparison of photocatalytic hydrogen evolution rates. Reprinted with permission from Ref. [128]. Copyright 2018, American Chemical Society

As a clean and efficient energy source, hydrogen is a promising alternative to fossil fuels. Therefore, photocatalytic hydrogen evolution has become a research focus [127]. In 2018, Thomas et al. [128] prepared two β-ketoenamine-based COFs, **TP-EDDA-COF** and **TP-BDDA-COF**, containing ethynyl and diethynyl structures, respectively. During photocatalytic hydrogen evolution, the hydrogen production rate of **TP-BDDA-COF** containing diethynyl group (324 μmol/(h·g)) was ten times higher than that of **TP-EDDA-COF** containing ethynyl group (30 μmol/(h·g)) (Fig. 28), suggesting that increasing the conjugated structure in the COF skeleton can make the charge mobility higher and significantly improve the photocatalytic activity.

**Fig. 29:**
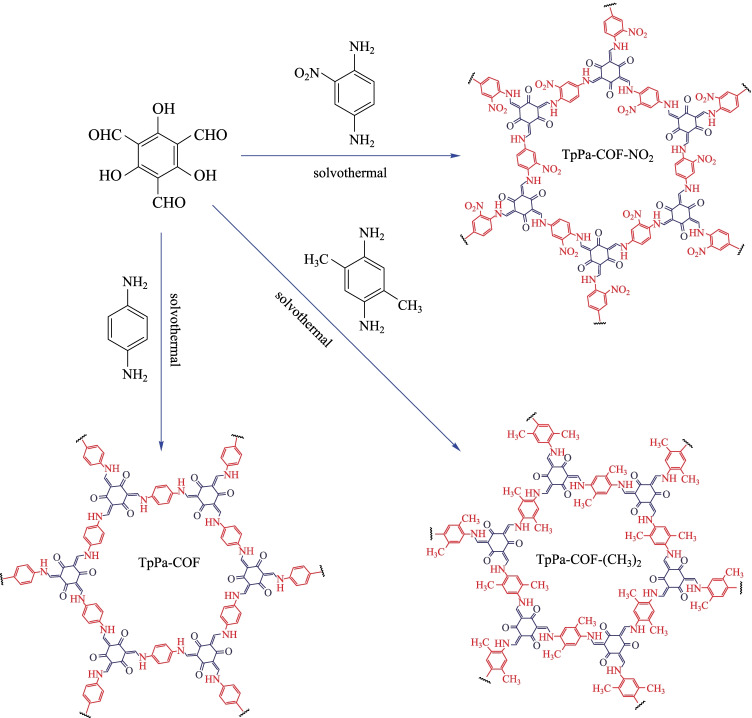
Synthesis of **TpPa-COF-X**. Reprinted with permission from Ref. [129]. Copyright 2019, WILEY–VCH Verlag GmbH & Co. KGaA

The electron donating or electron accepting units in the photocatalyst structure can also significantly improve the catalytic activity. The existence of the donor–acceptor unit can make the excited electrons transfer process more stable, to inhibit the recombination of excited electrons and holes. Sun et al. [129] prepared three β-ketoenamine-based COFs (**TpPa-COF**, **TpPa-COF-NO**_**2**_, **TpPa-COF-(CH**_**3**_**)**_**2**_) (Fig. 29). Under visible light irradiation, using platinum as a promoter and sodium ascorbate as a hole sacrificial agent, the hydrogen production rates were 1.56, 0.22, 8.33 mmol/(h·g), respectively. **TpPa-COF-(CH**_**3**_**)**_**2**_ containing electron donating units had certain catalytic activity while the electron-accepting units in **TpPa-COF-NO**_**2**_ or **TpPa-COF** without electron donor–acceptor unit showed almost no catalytic behavior. These results reveal that the introduction of electron-donating unit can improve the photocatalytic activity of the material, while the electron-withdrawing unit has the opposite effect. The reason for the opposite effect is that the electron-donating and electron-withdrawing groups have different conjugation effects, which affect the mobility of carriers and thus have different effects on the photocatalytic activity of the material.

**Fig. 30:**
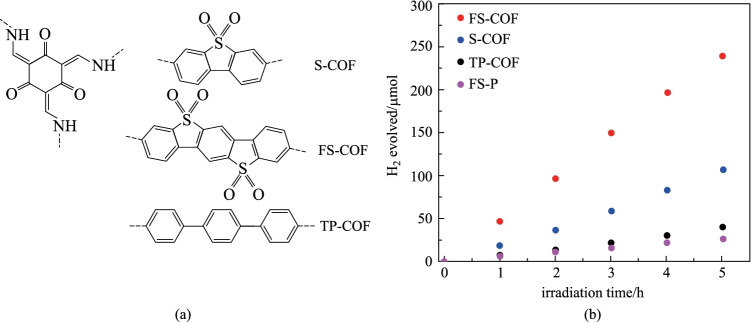
**a** Chemical structures of the COF: **S-COF**, **FS-COF** and **TP-COF**; **b** Time course for photocatalytic H_2_ production under visible light emission. Reprinted with permission from Ref. [130]. Copyright 2018, Springer Nature

In addition, researchers have also tried to introduce specific functional groups into the framework of COF materials to enhance the photocatalytic activity of COF materials. Cooper et al. [130] reported that sulfone-containing β-ketoenamine-based COFs for photocatalytic hydrogen evolution. **S-COF**, **FS-COF**, and **TP-COF** were respectively combined with 3,7-diaminodibenzo[b,d]thiophene sulfone (**SA**), 3,9-diamino-benzo[1,2-b:4,5-b′]bis [1]benzothiophene sulfone (**FSA**), 4,4′′-diamino-p-terphenyl (**TPA**) through reacting with the same monomer **TP** (Fig. 30a). Among them, compared with **S-COF** with one sulfone group and **TP-COF** without sulfone group, the hydrogen evolution rate of **FS-COF** with two sulfone groups was as high as 10.1 mmol/(h⋅g). Clearly, the number of sulfone groups has an important impact on the photocatalytic performance.

**Fig. 31:**
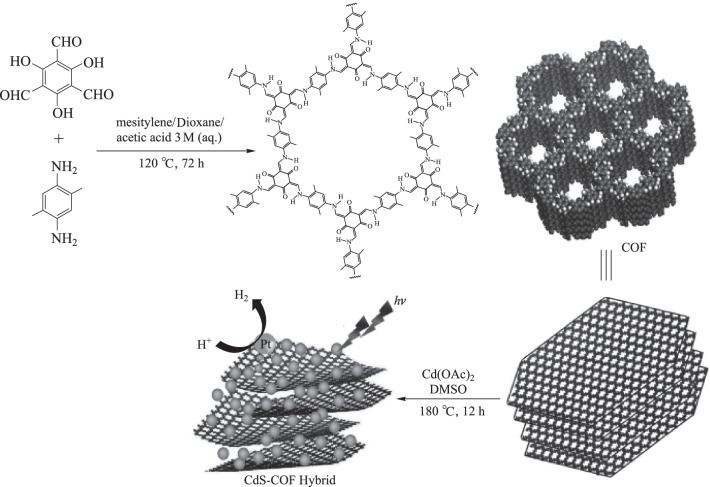
**CdS-COF** hybrid formation by hydrothermal synthesis of CdS nanoparticles on the COF matrix. Reprinted with permission from Ref. [131]. Copyright 2014, WILEY–VCH Verlag GmbH & Co. KGaA

Compared with activities of other photocatalytic materials, the COF catalytic activity of a single component is not high or the catalytic persistence is low. Therefore, COFs composites with other active semiconductors used to improve catalytic activity have been widely investigated. Banerjee et al. [131] first tried to combine β-ketoenamine-based COF (**TpPa-2**) with inorganic semiconductor CdS nanoparticles to prepare a photocatalytically active composite material **CdS@TpPa-2** (Fig. 31). Under light conditions, when the ratio of the two components (CdS:COFs) is 90:10, the efficiency of photolysis for hydrogen production could reach as high as 3678 μmol/(g·h), which is higher than that of CdS nanoparticles alone (124 μmol/(g·h)). The reason is that the uniform and ordered pore structure of **TpPa-2** promotes charge transfer and inhibits the transfer of photo-generated carriers and holes.

**Fig. 32:**
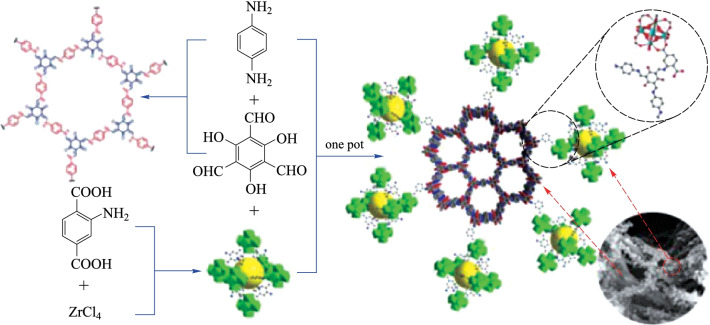
Preparation of **NH**_**2**_**-UiO-66/TpPa-1-COF** hybrid material. Reprinted with permission from Ref. [132]. Copyright 2018, WILEY–VCH Verlag GmbH & Co. KGaA

Liu et al. [132] successfully combined COF and MOF heterogeneously to construct a COF@MOF composite. Through the classic Schiff-base reaction, **NH**_**2**_**-UiO-66 (Zr)** covalently anchored onto the surface of **TpPa-1-COF**, the as-prepared **NH**_**2**_**-UiO-66/TpPa-1-COF** composite possessed porous structure and large surface area (Fig. 32). The detailed studies showed that the optimized mass ratio between **NH**_**2**_**-UiO-66** and **TpPa-1-COF** was 4:6, where the hydrogen evolution rate reached the highest amount (23.41 mmol/(g⋅h)). Such ultra-high hydrogen evolution rate is attributed to the good matching band gap between **NH**_**2**_**-UiO-66** and **TpPa-1-COF**, and the effective charge separation at the interface of the hybrid material.

**Fig. 33:**
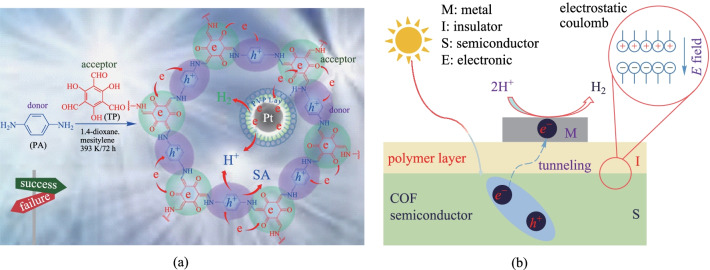
**a** Schematic illustration of **Pt-PVP-COFs** photosystems; **b** organic MIS nanostructured photocatalysts with a COF semiconductor assembled with polymer-capped Pt NPs. Reprinted with permission from Ref. [134]. Copyright 2019, WILEY–VCH Verlag GmbH & Co. KGaA

Similarly, graphitic carbon nitride (g-C_3_N_4_), as a polymeric organic semiconducting material, has also been used to prepare photocatalytic composites with COFs [133]. Under DMSO solvothermal conditions, **TP**, **TTA**, and g-C_3_N_4_ were condensed to prepare **CN-COF. TTA-TP-COF** is covalently connected to g-C_3_N_4_ through the imine bond between the terminal amino groups on the surface carbon nitride and the aldehyde groups in **TP**. The covalent bonds between g-C_3_N_4_ and COF facilitate the separation and the transfer of photogenerated carriers, thus enhancing the photocatalytic activity of the composite. Under visible light irradiation, the hydrogen evolution rate reached 10.1 mmol/(g·h). Recently, a new metal–insulator-semiconductor (MIS) nanostructure has been developed [134]. The hybrid photocatalytic **Pt-PVP-TP-COF** system was constructed using **TP-COF** as a semiconductor, polyvinylpyrrolidone **PVP** as an insulating layer, and Pt as a metal detector (Fig. 33a). The photoelectrons on the **TP-COF** could pass through the PVP insulating layer in the form of thermal tunneling to reach the metal detector. Under the irradiation of 475 nm wavelength light, the maximum hydrogen evolution rate reached 8.42 mmol/(g⋅h), and the maximum apparent quantum efficiency reached 0.4%. The results suggest that the MIS nanostructure cannot only enhance the light excitation and charge separation of the semiconductor part, but also promote the holes oxidize and accelerate the HER reaction.

#### Photocatalytic CO_2_ reduction

**Fig. 34:**
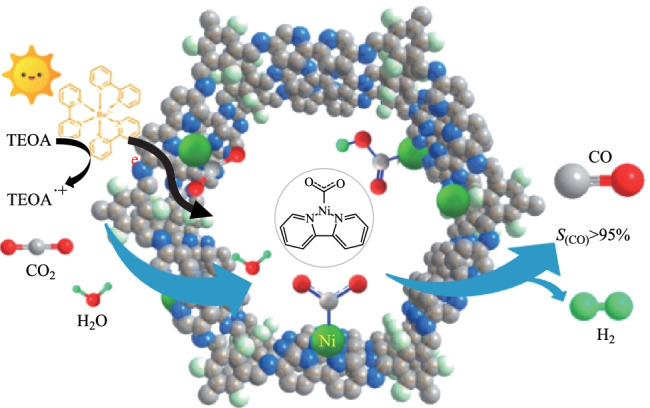
Schematic diagram photocatalytic selective reduction of CO_2_ over **Ni-TpBpy**. Reprinted with permission from Ref. [135]. Copyright 2019, American Chemical Society

Because of the excellent performance of β-ketoenamine-based COF hybrid materials in photocatalytic hydrogen evolution, researchers have explored their photocatalytic degradation performance in other directions such as photocatalytic CO_2_ reduction and photocatalytic degradation of organic pollutants. For example, Zou et al. [135] manufactured a nickel-containing 3D COF to selectively reduce CO_2_ to CO, where 2,2-bipyridyl COF was a carrier and Ni nanoparticles were highly dispersed on the surface of the COF pores (Fig. 34). The as-prepared COF composite (**Ni-TpBpy**) exhibited high CO generation activity (4057 μmol/g) within 5 h under water-containing conditions, and had a higher selectivity (96%) than hydrogen evolution (170 μmol/g). **Ni-TpBpy** showed a good conversion number (13.62) and quantum efficiency (0.3%). In addition, the catalyst could be reused up to three times, with a slight loss of catalytic activity. No major morphological and structural changes were observed.

**Fig. 35:**
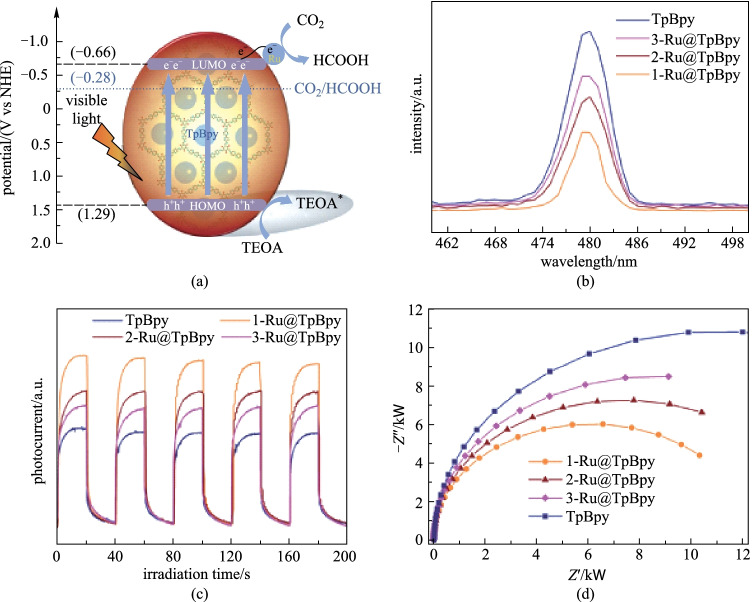
**a** Schematic diagram for photocatalytic CO_2_ reduction; **b** photoluminescence spectra; **c** photocurrent responses; **d** EIS Nyquist plots. Reprinted with permission from Ref. [136]. Copyright 2021, Elsevier

Fan et al. [136] built **Ru@TpBpy** composites by anchoring ruthenium nanoparticles (NPs) in a bipyridine-linked covalent organic framework (**TpBpy**). **Ru@TpBpy** catalyst can reduce CO_2_ to HCOOH under visible light irradiation (Fig. 35). The interaction between Ru-NPs and **TpBpy** cannot only enhance visible light capture, but also can effectively inhibit the recombination of photo-generated charges, promote electron transfer, and increase light catalytic CO_2_ reduction. The maximum yield of HCOOH reached 172 μmol/(g⋅h). Afterwards, in the same way, the authors prepared **Ru/TpPa-1** composites by anchoring ruthenium nanoparticles (NPs) onto β-ketoenamine- based COF **TpPa-1** [137]. Compared with the parent **TpPa-1**, **Ru/TpPa-1** photocatalyst showed a significantly enhanced CO_2_ reduction activity, and the maximum HCOOH yield was 108.8 μmol/(g⋅h).

#### Photocatalytic degradation

Giesy et al. [138] combined **TP** with **MA** at room temperature to form **TpMA** through mechanical ball milling. Under visible light irradiation of **TpMA**, the degradation rate of phenol reached 87.6%. In addition, Qin et al. [139] reported that **COF TpSD** containing sulfone bonds could be used as a photocatalyst to degrade organic dyes in water. Under the visible light of the xenon lamp, the rhodamine B (RhB) solution containing **COF TpSD** could be degraded completely within 30 min.

### Electrocatalysis

#### Electrocatalytic hydrogen generation

**Fig. 36:**
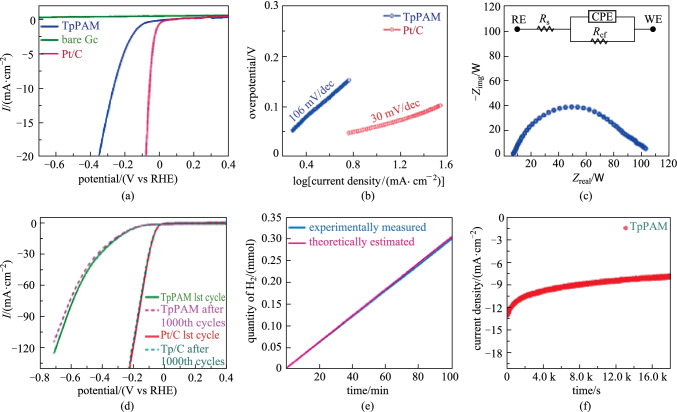
Electrocatalytic HER activity of the **TpPAM**. **a** LSV plots of **TpPAM**, Pt/C, and bare GC; **b** Tafel plots of **TpPAM** and Pt/C; **c** Nyquist plot of the **TpPAM** modified electrode; **d** LSV plots of **TpPAM**; **e** Faradaic efficiency for H_2_ generation; **f** Chronoamperometry (*I*−*t*) plot. Reprinted with permission from Ref. [140]. Copyright 2017, American Chemical Society

Porphyrin-based COF (**TpPAM**) [140] have been prepared by the condensation of **TP** and 5, 10, 15, 20-tetra(4-aminophenyl)-21H, 23H-porphyrin (**PAM**). As shown in Fig. 36, the as-prepared **TpPAM** COF exhibited superior activity for the electrocatalytic HER, with a relatively low overpotential (250 mV) and a small Tafel slope (106 mV/dec). The current density reached 10 mA/cm^2^. The Faradaic efficiency of the **TpPAM** based electrode was calculated to be 98%, which is very close to the ideal value of 100%. Furthermore, the as-synthesized catalyst showed good stability (retaining 91% of current density after 1000 cycles).

#### Electrocatalytic oxygen evolution

Electrochemical reaction is a convenient way to realize the effective interconversion of chemical energy and electric energy through bond breaking and formation. This method can split water electrochemically into hydrogen and oxygen, which can react with each other to regenerate water for the cycling realization of the decomposition and transformation of water [141]. The whole process is renewable, clean and green. In the two half reactions, the reducing hydrogen evolution reaction (HER) is faster, while the oxygen evolution reaction (OER) is hindered by the complexity of the following process: (i) Breaking of OH bonds; (ii) The decomposition of water molecules and the formation of electron pairs; and (iii) The formation of energy-intensive O=O bonds. β-ketoenamine-based COFs are one class of ultra-stable COFs that can be coordinated with transition metals as effective oxygen evolution catalysts.

**Fig. 37:**
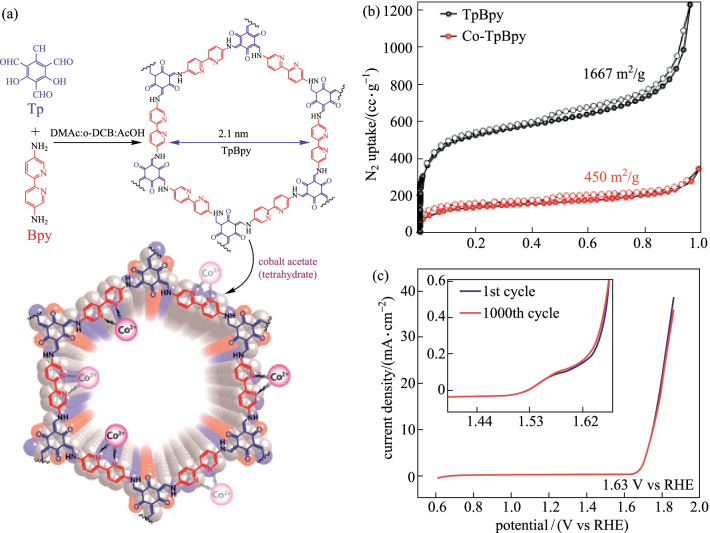
**a** Preparation of **TpBpy**; **b** N_2_ adsorption isotherms; **c** linear sweep voltammetry (LSV) stability test profile. Reprinted with permission from Ref. [142]. Copyright 2016, American Chemical Society

Banerjee et al. [142] synthesized bipyridine-containing COF by reacting **TP** with **BPY** (Fig. 37a). The bipyridine part can be used to coordinate with cobalt ions to generate abundant Co–N active sites and construct a metal-bipyridine molecular system. Figure 37b shows that the *S*_BET_ of **Co-TpBpy** was 450 m^2^/g, which wass less than that of **TpBpy** (1667 m^2^/g). In addition, **Co-TpBpy** showed remarkable LSV stability because its OER current retention rate was as high as 94% even after 1000 scans from 0.6 to 1.8 V (Fig. 37c). Under neutral pH conditions, the current density was 1 mA/cm^2^ and the overpotential was 400 mV.

**Fig. 38:**
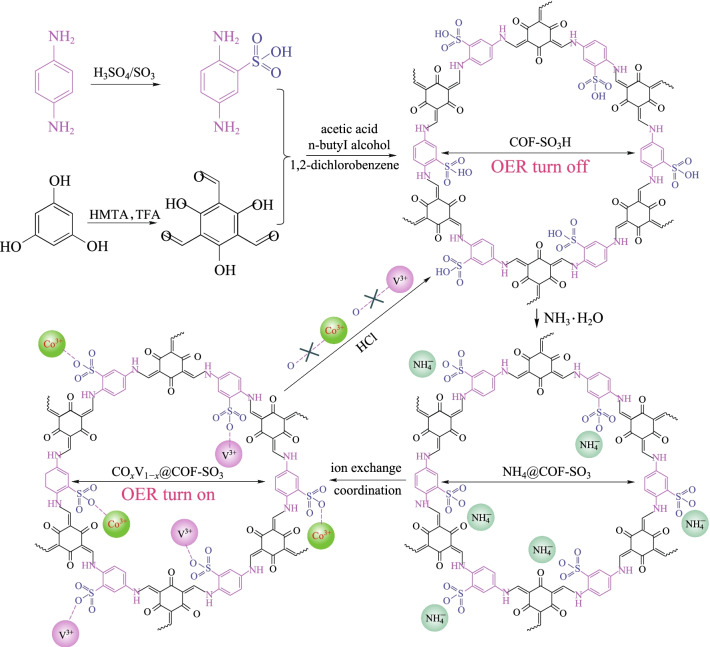
Strategy using a flexible and robust bimetal-incorporated COF catalyst to enable switching OER performance. Reprinted with permission from Ref. [143]. Copyright 2020, The Royal Society of Chemistry

Recently, a bipyridyl-containing COF loaded with bimetal (Co-V) has been synthesized for electrocatalytic oxygen evolution [143]. As shown in Fig. 38, the hybrid material is modified by − SO_3_H, followed by treatment with NH_4_^+^ and exchanged with cations. The bimetallic ions were successfully anchored to a specific site. When the bimetal ratio was 1:1 (i.e., **Co**_**0.5**_**V**_**0.5**_**@COF-SO**_**3**_), it showed the best electrocatalytic oxygen evolution activity. When the overpotential increased to 400 mV, a current density as high as 119.6 mA/cm^2^ could be obtained. The current density of **Co0.5V0.5@COF-SO**_**3**_ was approximately 6.2 times than that of vanadium-free **Co@COF-SO**_**3**_ (19.4 mA), indicating that V incorporation can significantly enhance OER activity. It is worth noting that in this system, bimetallic ions could be easily removed by HCl to ammoniate the phase for the next run to exchange with bimetallic ions, thereby realizing the reversible switching from the catalytically inert phase to the catalytically active phase.

**Fig. 39:**
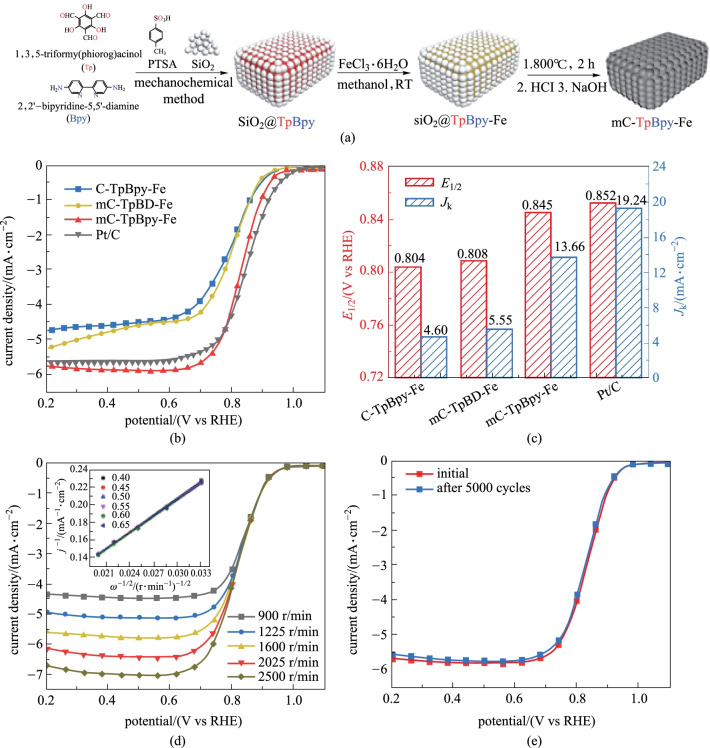
**a** Preparation of **mc-TpBpy-Fe**; **b** LSV curves; **c** half-wave potential and kinetic current; **d** LSV curves of **mC-TpBpy-Fe**; **e** LSV of **mC-TpBpy-Fe** before and after 5000 cycles at a voltage range between 0.7 and 1.0 V. Reprinted with permission from Ref. [145]. Copyright 2019, American Chemical Society

In 2019, Thomas et al. [144] reported on a template-assisted method to prepare β-ketoenamine- based COFs with hierarchical macroporous and microporous structures. Polystyrene spheres (PSs) were used as hard templates to form interconnected large pores within the COF structure. In particular, the size of the macropores could be adjusted by tuning the size of PSs, and the composition could be changed by using different COF precursors. The as-obtained macroporous COF maintained high crystallinity, high specific surface area, hierarchical pore structure, and chemically-stable framework, thus leading to rapid mass transfer and more accessible active sites. The use of cobalt-coordinated bipyridyl COF as the OER catalyst confirmed the rapid mass and ion transport in the hierarchical COF structure. The as-synthesized **macro-TpBpy-Co** showed excellent performance as an OER catalyst. Compared with pure microporous COF, its competitive overpotential at 10 mA/cm was 380 mV, and the surface was larger. The introduction of pore structure greatly improved the performance of electrocatalytic oxygen evolution. Therefore macroporous and microporous COF containing Fe (**mc-TpBpy-Fe**) [145] was prepared through a PTSA-assisted mechanochemical method in the presence of silica nanoparticles (Fig. 39a). The bipyridine parts of COF contributed to the coordination of metal ions and produced abundant Fe–N active sites in the resulting carbon material. The mesoporous structure and macroporous volume effectively promoted the diffusion of O_2_ and the electrolyte into the uniformly dispersed FefiNx active centers, resulting in excellent ORR performance with a competitive half-wave potential of 0.845 V and a limiting current density of 5.92 mA/cm^2^.

### Batteries

**Fig. 40:**
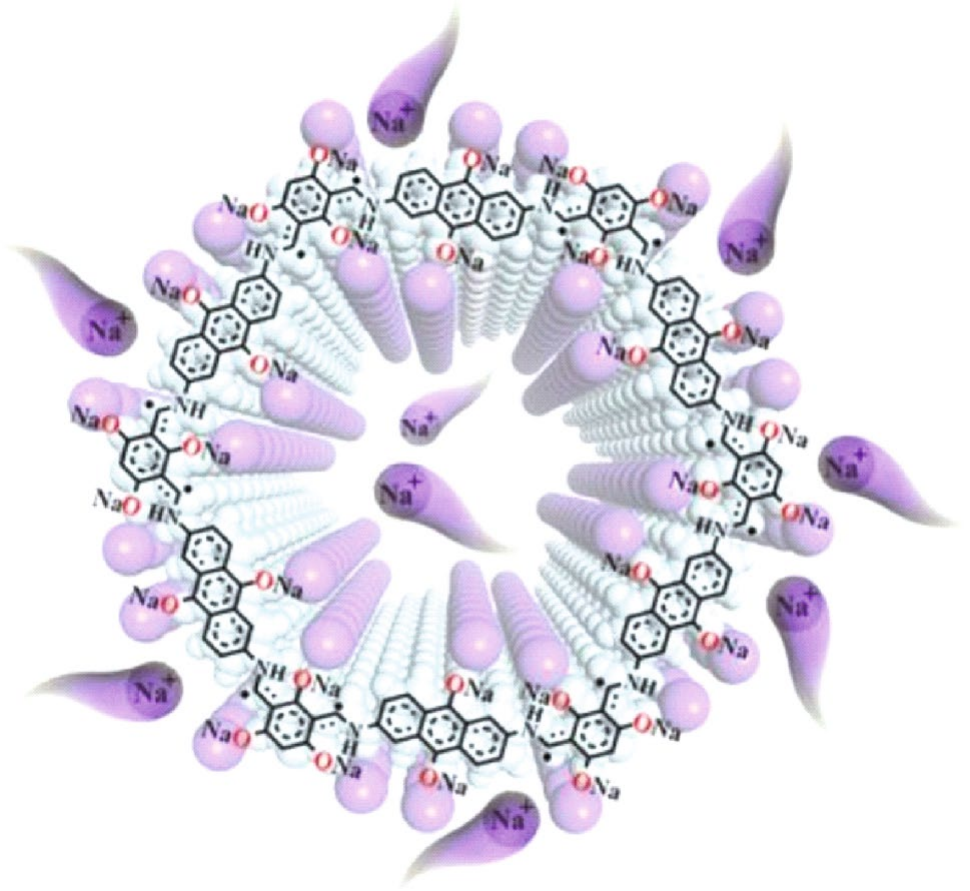
Sodium ion transmission in 2D COF. Reprinted with permission from Ref. [146]. Copyright 2019, American Chemical Society

There are few reports on the use of β-ketoenamine-based COFs as electrodes in rechargeable batteries. Lu et al. [146] reported a high-capacity stable anode based on a β-ketoenamine COF in a sodium-ion battery (Fig. 40). The characterization revealed that the COF electrode promoted the formation and transformation of C-O and α-C free radical intermediates in the redox process. The results indicated that the stacking interaction was essential for controlling electrochemical performance. Reducing the stacking thickness of 2D COF could systematically and simultaneously improve the stability of free radical intermediates. A 4–12 nm thickness of 2D-COF samples showed very promising electrochemical performance with high capacity (420 mAh/g at 100 mA/g), excellent rate performance (at 5 A/g, 198 mAh/g) and excellent cycle stability (99% retention rate in 10000 cycles at 5 A/g).

### Proton conduction

**Fig. 41:**
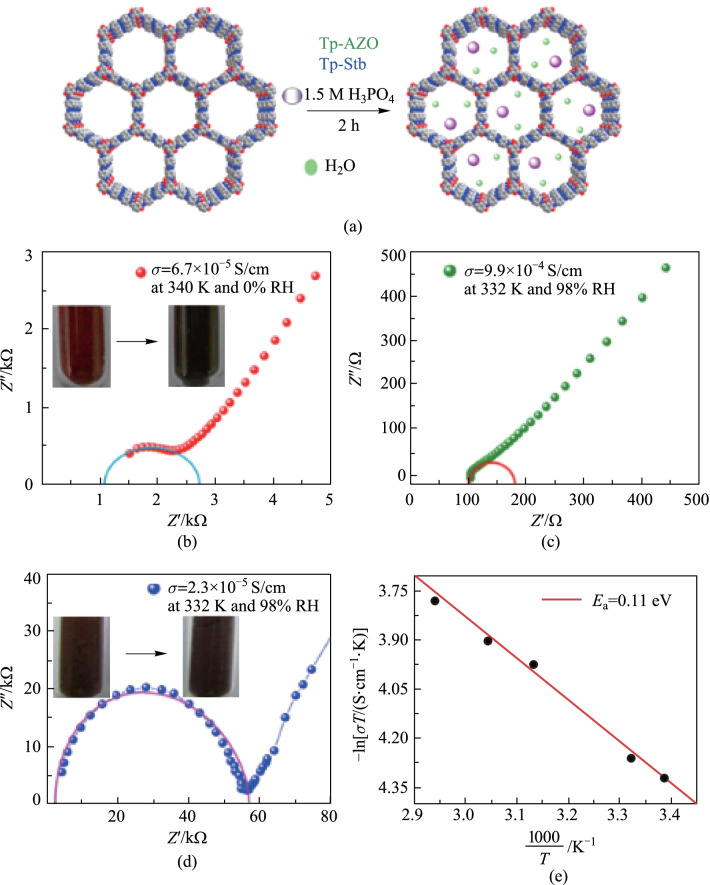
**a** Schematic of COF doping by H_3_PO_4_; **b** anhydrous and **c** hydrous conditions. **d** Proton conductivity of **PA@Tp-Stb**; **e** Arrhenius plot in hydrous conditions with **PA@Tp-Azo**. Reprinted with permission from Ref. [147]. Copyright 2014, American Chemical Society

In recent years, proton conductive materials have attracted great interest due to their wide applications in fuel cells, sensors and electronic devices. The high stability of β-ketoenamine-based COFs ensure the sustainability of materials under harsh fuel cell operating conditions. As shown in Fig. 41, in 2014, Benerjee et al. [147] proposed an azo-functionalized COF (**Tp-Azo**) containing a Schiff-base between those of **TP** and **AZO**. Doping H_3_PO_4_ in **Tp-Azo** results in acid immobilization in the porous framework and the as-prepared **PA@Tp-Azo** shows decent proton conductivity under humid and anhydrous conditions (9.9 × 10^−4^ and 6.7 × 10^−5^ S/cm, respectively).

**Fig. 42:**
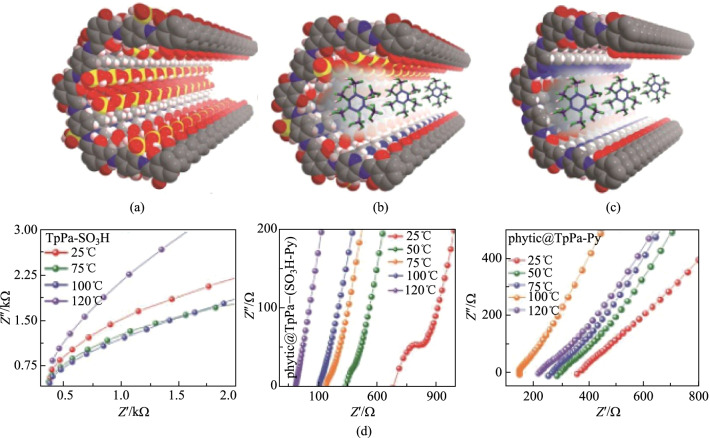
**a TpPa-SO**_**3**_**H; b** **phytic@TpPa-(SO**_**3**_**H-Py)**; **c** **phytic@TpPa-Py**; **d** their corresponding proton conductivities with Nyquist plots. Reprinted with permission from Ref. [148]. Copyright 2016, American Chemical Society

Furthermore, the intrinsic and extrinsic proton conduction phenomena in COFs can be realized in two ways [148], where sulfonic acid-functionalized COF (**TpPa-SO**_**3**_**H**) was severed as an intrinsic proton conductor (Fig. 42a). Free −SO_3_H groups were stacked in the COF backbone to provide a continuous array of proton conduction sites. Afterwards, a pyridine functionalized COF was synthesized by integrating a pyridyl site (**TpPa-Py**) and a hybrid COF containing pyridyl and sulfonic acid groups **TpPa-(SO**_**3**_**H-Py)** VERB into one COF structure via a ligand-based solid solution method. Subsequently, phytic acid phytate (sodium dihydrogen phosphate) was immersed in **TpPa Py** and **TpPa-(SO**_**3**_**H Py)** to form **phytic@TpPa-Py** and **phytic@TpPa-(SO**_**3**_**H-Py)**, respectively. **Phytic@TpPa-(SO**_**3**_**H-Py)** could conduct protons through the combination of sulfonic acid groups with adjacent pyridine units (intrinsically), or transport protons through immobilized phytic acid at the pyridine site (externally). The anhydrous proton conductivity of **phytic@TpPa-(SO**_**3**_**H-Py)** (5 × 10^−4^ S/cm at 120 °C) was higher than those of **phytic@TpPa-Py** and **phytic@TpPa-SO**_**3**_**H** (Fig. 42b).

**Fig. 43:**
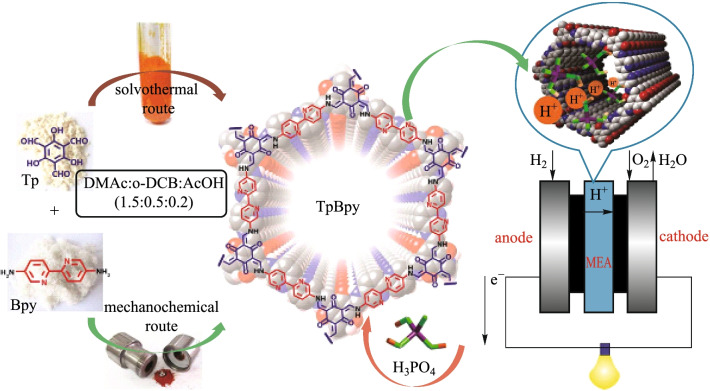
Synthesis of **TpBpy COF** [via mechanochemical (MC) and solvothermal (ST) route] as well as the loading of phosphoric acid (PA) forms **PA@TpBpy** and the integration as solid electrolyte in PEMFCs. Reprinted with permission from Ref. [87]. Copyright 2016, The Royal Society of Chemistry

As shown in Fig. 43, Benerjee et al. [87] reported that **TP** and **BPY** were combined together by the solvothermal method (**TpBpy ST**) or by mechanochemical method (**TpBpy MC**). Diamine (**BPY**) bipyridine-functionalized COF was synthesized through the Schiff-base reaction. Compared with **TpBpy-ST-COFs**, **TpBpy-MC** had lower crystallinity and small porosity. However, these characteristics could ensure **TpBpy-MC** offered a high-efficiency solid electrolyte (proton conductivity of 1.4 × 10^−2^ S/cm) in PEM fuel cells, while the solvothermally synthesized COF under similar conditions showed no proton conductivity.

## Summary and outlook

β-Ketoenamine-based COFs are obtained by irreversible enol-to-keto tautomerization from imine-based COFs, which exhibit excellent stability, ultra-high specific surface area, and adjustable pore size. In this mini review, different synthetic methods such as solvothermal, mechanochemical, microwave, ionthermal and hydrothermal synthesis are summarized. The characteristics of different morphologies of β-Ketoenamine-based COFs such as powder, film and foam are also introduced and compared. The optoelectrical applications of β-Ketoenamine-based COFs including fluorescence detection, energy storage, photocatalysis, electrocatalysis, batteries, and protons conductor etc., are also presented.

In fact, most β-ketoenamine-based COFs are synthesized by the solvothermal method. However, the synthetized amount can only reach the milligram level. Although the reported room-temperature mechanical grinding method can prepare gram-scale β-ketoenamine-based COFs, the crystallinity and porosity of the as-synthesized COFs are not satisfactory. Microwave-assisted method can save time and generate high quality crystalline material. However, organic solvents are required, which leads to a large number of VOCs. Hydrothermal synthesis is a green method; however, it requires relatively high temperature and long reaction time. As to the further development of β-ketoenamine-based COFs, a large-scale, low-cost, and green synthetic method is highly desirable in the future. Moreover, although β-ketoenamine-based COFs have been applied in many fields, they have not been applied in biological field. In fact, β-ketoenamine-based COFs not only contain a large number of hydrophilic groups such as C=O, OH and NH, but also display excellent optoelectrical properties, which might make these COFs promising candidates for potential applications in bioimaging, photothermal and photodynamic cancer therapy etc. in the future.

## Footnotes

1 1 M = 1 mol/L.

## References

[1] Liu X, Huang D, Lai C, Zeng G, Qin L, Wang H, Yi H, Li B, Liu S, Zhang M, Deng R, Fu Y, Li L, Xue W, Chen S (2019). Recent advances in covalent organic frameworks (COFs) as a smart sensing material. Chem. Soc. Rev..

[2] Yusran Y, Fang Q, Qiu SL (2018). Postsynthetic covalent modification in covalent organic frameworks. Isr. J. Chem..

[3] Gui B, Lin G, Ding H, Gao C, Mal A, Wang C (2020). Three-dimensional covalent organic frameworks: from topology design to applications. Acc. Chem. Res..

[4] Alahakoon SB, Diwakara SD, Thompson CM, Smaldone RA (2020). Supramolecular design in 2D covalent organic frameworks. Chem. Soc. Rev..

[5] Wang X, She P, Zhang Q (2021). Recent advances on electrochemical methods in fabricating two-dimensional organic-ligand-containing frameworks. SmartMat.

[6] Xu S, Zhang QC (2021). Recent progress in covalent organic frameworks as light-emitting materials. Mater. Today. Energy.

[7] Li Y, Chen W, Xing G, Jiang D, Chen L (2020). New synthetic strategies toward covalent organic frameworks. Chem. Soc. Rev..

[8] Wang Z, Zhang S, Chen Y, Zhang Z, Ma S (2020). Covalent organic frameworks for separation applications. Chem. Soc. Rev..

[9] Dautzenberg E, Lam M, Li G, de Smet LCPM (2021). Enhanced surface area and reduced pore collapse of methylated, imine-linked covalent organic frameworks. Nanoscale.

[10] She, P., Qin, Y., Wang, X., Zhang, Q.: Recent progress in external-stimulus-responsive 2D covalent organic frameworks. Adv. Mater.** 34**(22), 2101175 (2021) 10.1002/adma.202101175 34240479

[11] Yang J, Kang F, Wang X, Zhang Q (2022). Design strategies for improving the crystallinity of covalent organic frameworks and conjugated polymers: a review. Mater. Horiz..

[12] Zhan XJ, Chen Z, Zhang QC (2017). Recent progress in two-dimensional COFs for energy-related applications. J. Mater. Chem. A Mater. Energy Sustain..

[13] Wu C, Liu Y, Liu H, Duan C, Pan Q, Zhu J, Hu F, Ma X, Jiu T, Li Z, Zhao Y (2018). Highly conjugated three-dimensional covalent organic frameworks based on spirobifluorene for perovskite solar cell enhancement. J. Am. Chem. Soc..

[14] Sun T, Xie J, Guo W, Li DS, Zhang QC (2020). Covalent–organic frameworks: advanced organic electrode materials for rechargeable batteries. Adv. Energy Mater..

[15] Yao CJ, Wu Z, Xie J, Yu F, Guo W, Xu ZJ, Li DS, Zhang S, Zhang Q (2020). Two-dimensional (2D) covalent organic framework as efficient cathode for binder-free lithium-ion battery. Chemsuschem.

[16] Bagheri AR, Aramesh N, Haddad PR (2022). Applications of covalent organic frameworks and their composites in the extraction of pesticides from different samples. J. Chromatogr. A.

[17] Zhou HY, Zhou CD, Tang SY, Zhang FM, Lei S, Li ZJ, Liu JJ, Chen M (2022). High efficiency solution synthesis of aryl-aryl linked two-dimensional covalent organic frameworks with remarkable adsorption performance for polycyclic aromatic hydrocarbons (PAHs). Mater. Lett..

[18] He M, Liang QH, Tang L, Liu ZF, Shao BB, He QY, Wu T, Luo SH, Pan Y, Zhao CH, Niu CG, Hu YM (2021). Advances of covalent organic frameworks based on magnetism: classification, synthesis, properties, applications. Coord. Chem. Rev..

[19] Wang H, Wang MK, Wang YL, Wang J, Men XH, Zhang ZZ, Singh V (2021). Synergistic effects of COF and GO on high flux oil/water separation performance of superhydrophobic composites. Sep. Purif. Technol..

[20] Yu F, Liu W, Ke SW, Kurmoo M, Zuo JL, Zhang Q (2020). Electrochromic two-dimensional covalent organic framework with a reversible dark-to-transparent switch. Nat. Commun..

[21] Yu F, Liu W, Li B, Tian D, Zuo JL, Zhang Q (2019). Photostimulus-responsive large-area two-dimensional covalent organic framework films. Angew. Chem. Int. Ed..

[22] Xiang Y, Yu XL, Li YQ, Chen JY, Wu JJ, Wang LX, Chen DG, Li JB, Zhang QC (2021). Covalent organic framework as an efficient fluorescence-enhanced probe to detect aluminum ion. Dyes Pigm..

[23] Yao S, Liu Z, Li L (2021). Recent progress in nanoscale covalent organic frameworks for cancer diagnosis and therapy. Nano-Micro Letters.

[24] Ghahari A, Raissi H, Farzad F (2021). Design of a new drug delivery platform based on surface functionalization 2D covalent organic frameworks. J. Taiwan Inst. Chem. Eng..

[25] Gao P, Shen X, Liu X, Chen Y, Pan W, Li N, Tang B (2021). Nucleic acid-gated covalent organic frameworks for cancer-specific imaging and drug release. Anal. Chem..

[26] Zhi Y, Wang Z, Zhang HL, Zhang Q (2020). Recent progress in metal-free covalent organic frameworks as heterogeneous catalysts. Small.

[27] Sun QZ, Wu CY, Pan QY, Zhang BJ, Liu YM, Lu XY, Sun J, Sun LS, Zhao YJ (2021). Three-dimensional covalent-organic frameworks loaded with highly dispersed ultrafine palladium nanoparticles as efficient heterogeneous catalyst. ChemNanoMat.

[28] Chen Y, Li W, Wang X, Gao R, Tang A, Kong D (2021). Green synthesis of covalent organic frameworks based on reaction media. Mater. Chem. Front..

[29] Maia RA, Lopes Oliveira F, Ritleng V, Wang Q, Louis B, Mothé EP (2021). CO_2_ capture by hydroxylated azine-based covalent organic frameworks. Chemistry (Weinheim an der Bergstrasse, Germany).

[30] Haase F, Lotsch BV (2020). Solving the COF trilemma: towards crystalline, stable and functional covalent organic frameworks. Chem. Soc. Rev..

[31] Fan C, Wu H, Guan J, You X, Yang C, Wang X, Cao L, Shi B, Peng Q, Kong Y, Wu Y, Khan NA, Jiang Z (2021). Scalable fabrication of crystalline COF membrane from amorphous polymeric membrane. Angew. Chem. Int. Ed..

[32] Dawson R, Cooper AI, Adams DJ (2012). Nanoporous organic polymer networks. Prog. Polym. Sci..

[33] Zhu YL, Zhao HY, Fu CL, Li ZW, Sun ZY, Lu Z (2020). Mechanisms of defect correction by reversible chemistries in covalent organic frameworks. J. Phys. Chem. Lett..

[34] Mao CF, Hu YJ, Yang CH, Qin CC, Dong GM, Zhou YM, Zhang YW (2021). Well-designed spherical covalent organic frameworks with an electron-deficient and conjugate system for efficient photocatalytic hydrogen evolution. ACS Appl. Energy Mater..

[35] Hynek J, Zelenka J, Rathouský J, Kubát P, Ruml T, Demel J, Lang K (2018). Designing porphyrinic covalent organic frameworks for the photodynamic inactivation of bacteria. ACS Appl. Mater. Interfaces..

[36] Meng F, Bi S, Sun Z, Jiang B, Wu D, Chen JS, Zhang F (2021). Synthesis of ionic vinylene-linked covalent organic frameworks through quaternization-activated knoevenagel condensation. Angew. Chem. Int. Ed..

[37] Segura JL, Mancheño MJ, Zamora F (2016). Covalent organic frameworks based on Schiff-base chemistry: synthesis, properties and potential applications. Chem. Soc. Rev..

[38] Cote AP, Benin AI, Ockwig NW, O'Keeffe M, Matzger AJ, Yaghi OM (2005). Porous, crystalline, covalent organic frameworks. Science.

[39] Lanni LM, Tilford RW, Bharathy M, Lavigne JJ (2011). Enhanced hydrolytic stability of self-assembling alkylated two-dimensional covalent organic frameworks. J. Am. Chem. Soc..

[40] Park S, Liao Z, Ibarlucea B, Qi H, Lin HH, Becker D, Melidonie J, Zhang T, Sahabudeen H, Baraban L, Baek CK, Zheng Z, Zschech E, Fery A, Heine T, Kaiser U, Cuniberti G, Dong R, Feng X (2020). Two-dimensional boronate ester covalent organic framework thin films with large single crystalline domains for a neuromorphic memory device. Angew. Chem. Int. Ed..

[41] Li YJ, Han YN, Chen MH, Feng YQ, Zhang B (2019). Construction of a flexible covalent organic framework based on triazine units with interesting photoluminescent properties for sensitive and selective detection of picric acid. RSC Adv..

[42] Li XL, Cai SL, Sun B, Yang CQ, Zhang J, Liu Y (2020). Chemically robust covalent organic frameworks: progress and perspective. Matter.

[43] Lu Y, Liang Y, Zhao Y, Xia M, Liu X, Shen T, Feng L, Yuan N, Chen Q (2021). Fluorescent test paper via the in situ growth of COFs for rapid and convenient detection of Pd(II) ions. ACS Appl. Mater. Interfaces..

[44] Shan M, Seoane B, Rozhko E, Dikhtiarenko A, Clet G, Kapteijn F, Gascon J (2016). Azine-linked covalent organic framework (COF)-based mixed-matrix membranes for CO_2_/CH_4_ separation. Chemistry (Weinheim an der Bergstrasse, Germany).

[45] Luo Z, Liu L, Ning J, Lei K, Lu Y, Li F, Chen J (2018). A microporous covalent-organic framework with abundant accessible carbonyl groups for lithium-ion batteries. Angew. Chem. Int. Ed..

[46] Xue R, Gou H, Zheng YP, Zhang L, Liu YS, Rao HH, Zhao GH (2020). A new squaraine-linked triazinyl-based covalent organic frameworks: preparation, characterization and application for sensitive and selective determination of Fe^3+^ cations. ChemistrySelect.

[47] Wei S, Zhang F, Zhang W, Qiang P, Yu K, Fu X, Wu D, Bi S, Zhang F (2019). Semiconducting 2D triazine-cored covalent organic frameworks with unsubstituted olefin linkages. J. Am. Chem. Soc..

[48] Yuan C, Fu S, Yang K, Hou B, Liu Y, Jiang J, Cui Y (2021). Crystalline C–C and C═C bond-linked chiral covalent organic frameworks. J. Am. Chem. Soc..

[49] Cusin L, Peng H, Ciesielski A, Samorì P (2021). Chemical conversion and locking of the imine linkage: enhancing the functionality of covalent organic frameworks. Angew. Chem. Int. Ed..

[50] Han X, Huang J, Yuan C, Liu Y, Cui Y (2018). Chiral 3D covalent organic frameworks for high performance liquid chromatographic enantioseparation. J. Am. Chem. Soc..

[51] Liu HY, Chu J, Yin ZL, Cai X, Zhuang L, Deng HX (2018). Covalent organic frameworks linked by amine bonding for concerted electrochemical reduction of CO_2_. Chem.

[52] Liang Y, Xia M, Zhao Y, Wang D, Li Y, Sui Z, Xiao J, Chen Q (2022). Functionalized triazine-based covalent organic frameworks containing quinoline via aza-Diels-Alder reaction for enhanced lithium-sulfur batteries performance. J. Colloid Interface Sci..

[53] Rabbani MG, El-Kaderi HM (2012). Synthesis and characterization of porous benzimidazole-linked polymers and their performance in small gas storage and selective uptake. Chem. Mater..

[54] Rabbani MG, Islamoglu T, El-Kaderi HM (2017). Benzothiazole- and benzoxazole-linked porous polymers for carbon dioxide storage and separation. J. Mater. Chem. A Mater. Energy Sustain..

[55] Waller PJ, AlFaraj YS, Diercks CS, Jarenwattananon NN, Yaghi OM (2018). Conversion of imine to oxazole and thiazole linkages in covalent organic frameworks. J. Am. Chem. Soc..

[56] Eder GM, Pyles DA, Wolfson ER, McGrier PL (2019). A ruthenium porphyrin-based porous organic polymer for the hydrosilylative reduction of CO_2_ to formate. Chem. Commun..

[57] Machado TF, Serra MES, Murtinho D, Valente AJM, Naushad M (2021). Covalent organic frameworks: synthesis, properties and applications-an overview. Polymers.

[58] Sharma RK, Yadav P, Yadav M, Gupta R, Rana P, Srivastava A, Zboril R, Varma RS, Antonietti M, Gawande MB (2020). Recent development of covalent organic frameworks (COFs): synthesis and catalytic (organic-electro-photo) applications. Mater. Horiz..

[59] Tao Y, Ji WY, Ding XS, Han BH (2021). Exfoliated covalent organic framework nanosheets. J. Mater. Chem. A Mater. Energy Sustain..

[60] Bai B, Wang D, Wan LJ (2021). Synthesis of covalent organic framework films at interfaces. Bull. Chem. Soc. Jpn..

[61] Mohammed AK, Shetty D (2021). Macroscopic covalent organic framework architectures for water remediation. Environ. Sci.: Water Res. Technol..

[62] Li X, Zhang C, Cai S, Lei X, Altoe V, Hong F, Urban JJ, Ciston J, Chan EM, Liu Y (2018). Facile transformation of imine covalent organic frameworks into ultrastable crystalline porous aromatic frameworks. Nat. Commun..

[63] Evans AM, Ryder MR, Ji W, Strauss MJ, Corcos AR, Vitaku E, Flanders NC, Bisbey RP, Dichtel WR (2021). Trends in the thermal stability of two-dimensional covalent organic frameworks. Faraday Discuss..

[64] Rao MR, Fang Y, De Feyter S, Perepichka DF (2017). Conjugated covalent organic frameworks via michael addition-elimination. J. Am. Chem. Soc..

[65] Babu HV, Bai MGM, Rajeswara RM (2019). Functional π-conjugated two-dimensional covalent organic frameworks. ACS Appl. Mater. Interfaces..

[66] Esrafili A, Wagner A, Inamdar S, Acharya AP (2021). Covalent organic frameworks for biomedical applications. Adv. Healthcare Mater..

[67] Singh V, Jang S, Vishwakarma NK, Kim D (2018). Intensified synthesis and post-synthetic modification of covalent organic frameworks using a continuous flow of microdroplets technique. NPG Asia Mater..

[68] Wang JL, Zhuang ST (2019). Covalent organic frameworks (COFs) for environmental applications. Coord. Chem. Rev..

[69] Chong JH, Sauer M, Patrick BO, MacLachlan MJ (2003). Highly stable keto-enamine salicylideneanilines. Org. Lett..

[70] Kandambeth S, Mallick A, Lukose B, Mane MV, Heine T, Banerjee R (2012). Construction of crystalline 2D covalent organic frameworks with remarkable chemical (acid/base) stability via a combined reversible and irreversible route. J. Am. Chem. Soc..

[71] Li X, Yang C, Sun B, Cai S, Chen Z, Lv Y, Zhang J, Liu Y (2020). Expeditious synthesis of covalent organic frameworks: a review. J. Mater. Chem. A Mater. Energy Sustain..

[72] Ji W, Hamachi LS, Natraj A, Flanders NC, Li RL, Chen LX, Dichtel WR (2021). Solvothermal depolymerization and recrystallization of imine-linked two-dimensional covalent organic frameworks. Chem. Sci. (Cambridge).

[73] Zou LF, Yang XY, Yuan S, Zhou HC (2017). Flexible monomer-based covalent organic frameworks: design, structure and functions. CrystEngComm.

[74] DeBlase CR, Silberstein KE, Truong TT, Abruña HD, Dichtel WR (2013). β-Ketoenamine-linked covalent organic frameworks capable of pseudocapacitive energy storage. J. Am. Chem. Soc..

[75] Fang Q, Gu S, Zheng J, Zhuang Z, Qiu S, Yan Y (2014). 3D microporous base-functionalized covalent organic frameworks for size-selective catalysis. Angew. Chem. Int. Ed..

[76] Wolfe JP, Åhman J, Sadighi JP, Singer RA, Buchwald SL (1997). An ammonia equivalent for the palladium-catalyzed amination of aryl halides and triflates. Tetrahedron Lett..

[77] Vitaku E, Dichtel WR (2017). Synthesis of 2D imine-linked covalent organic frameworks through formal transimination reactions. J. Am. Chem. Soc..

[78] Daugherty MC, Vitaku E, Li RL, Evans AM, Chavez AD, Dichtel WR (2019). Improved synthesis of β-ketoenamine-linked covalent organic frameworks via monomer exchange reactions. Chem. Commun..

[79] Wang R, Kong W, Zhou T, Wang C, Guo J (2021). Organobase modulated synthesis of high-quality β-ketoenamine-linked covalent organic frameworks. Chem. Commun..

[80] Zhao C, Diercks CS, Zhu C, Hanikel N, Pei X, Yaghi OM (2018). Urea-linked covalent organic frameworks. J. Am. Chem. Soc..

[81] Biswal BP, Chandra S, Kandambeth S, Lukose B, Heine T, Banerjee R (2013). Mechanochemical synthesis of chemically stable isoreticular covalent organic frameworks. J. Am. Chem. Soc..

[82] Chandra S, Kandambeth S, Biswal BP, Lukose B, Kunjir SM, Chaudhary M, Babarao R, Heine T, Banerjee R (2013). Chemically stable multilayered covalent organic nanosheets from covalent organic frameworks via mechanical delamination. J. Am. Chem. Soc..

[83] Liu W, Cao Y, Wang W, Gong D, Cao T, Qian J, Iqbal K, Qin W, Guo H (2019). Mechanochromic luminescent covalent organic frameworks for highly selective hydroxyl radical detection. Chem. Commun..

[84] Das G, Balaji Shinde D, Kandambeth S, Biswal BP, Banerjee R (2014). Mechanosynthesis of imine, β-ketoenamine, and hydrogen-bonded imine-linked covalent organic frameworks using liquid-assisted grinding. Chem. Commun..

[85] Liu SS, Liu QQ, Huang SZ, Zhang C, Dong XY, Zang SQ (2022). Sulfonic and phosphonic porous solids as proton conductor. Coord. Chem. Rev..

[86] Peng Y, Xu G, Hu Z, Cheng Y, Chi C, Yuan D, Cheng H, Zhao D (2016). Mechanoassisted synthesis of sulfonated covalent organic frameworks with high intrinsic proton conductivity. ACS Appl. Mater. Interfaces..

[87] Shinde DB, Aiyappa HB, Bhadra M, Biswal BP, Wadge P, Kandambeth S, Garai B, Kundu T, Kurungot S, Banerjee R (2016). A mechanochemically synthesized covalent organic framework as a proton-conducting solid electrolyte. J. Mater. Chem. A Mater. Energy Sustain..

[88] Karak S, Kandambeth S, Biswal BP, Sasmal HS, Kumar S, Pachfule P, Banerjee R (2017). Constructing ultraporous covalent organic frameworks in seconds via an organic terracotta process. J. Am. Chem. Soc..

[89] Wei H, Chai S, Hu N, Yang Z, Wei L, Wang L (2015). The microwave-assisted solvothermal synthesis of a crystalline two-dimensional covalent organic framework with high CO_2_ capacity. Chem. Commun..

[90] Xu L, Xu J, Shan BT, Wang XL, Gao CJ (2017). TpPa-2-incorporated mixed matrix membranes for efficient water purification. J. Membr. Sci..

[91] Dong B, Wang WJ, Pan W, Kang GJ (2019). Ionic liquid as a green solvent for ionothermal synthesis of 2D keto-enamine-linked covalent organic frameworks. Mater. Chem. Phys..

[92] Qiu JK, Wang HY, Zhao YL, Guan PX, Li ZY, Zhang HC, Gao HS, Zhang SJ, Wang JJ (2020). Hierarchically porous covalent organic frameworks assembled in ionic liquids for highly effective catalysis of C-C coupling reactions. Green Chem..

[93] Zhao LM, Liu HM, Du Y, Liang X, Wang WJ, Zhao H, Li WZ (2020). An ionic liquid as a green solvent for high potency synthesis of 2D covalent organic frameworks. New J. Chem..

[94] Thote J, BarikeAiyappa H, Rahul Kumar R, Kandambeth S, Biswal BP, Balaji Shinde D, Chaki Roy N, Banerjee R (2016). Constructing covalent organic frameworks in water *via* dynamic covalent bonding. Int. Union Crystallogr..

[95] Lu J, Lin F, Wen Q, Qi QY, Xu JQ, Zhao X (2019). Large-scale synthesis of azine-linked covalent organic frameworks in water and promoted by water. N. J. Chem..

[96] DeBlase CR, Hernández-Burgos K, Silberstein KE, Rodríguez-Calero GG, Bisbey RP, Abruña HD, Dichtel WR (2015). Rapid and efficient redox processes within 2D covalent organic framework thin films. ACS Nano.

[97] Wang R, Wei MJ, Wang Y (2020). Secondary growth of covalent organic frameworks (COFs) on porous substrates for fast desalination. J. Membr. Sci..

[98] Liu GH, Jiang ZY, Yang H, Li CD, Wang HJ, Wang MD, Song YM, Wu H, Pan FS (2019). High-efficiency water-selective membranes from the solution-diffusion synergy of calcium alginate layer and covalent organic framework (COF) layer. J. Membr. Sci..

[99] Kandambeth S, Biswal BP, Chaudhari HD, Rout KC, Kunjattu HS, Mitra S, Karak S, Das A, Mukherjee R, Kharul UK, Banerjee R (2017). Selective molecular sieving in self-standing porous covalent-organic-framework membranes. Adv. Mater..

[100] Dey K, Bhunia S, Sasmal HS, Reddy CM, Banerjee R (2021). Self-assembly-driven nanomechanics in porous covalent organic framework thin films. J. Am. Chem. Soc..

[101] Dey K, Pal M, Rout KC, Kunjattu HS, Das A, Mukherjee R, Kharul UK, Banerjee R (2017). Selective molecular separation by interfacially crystallized covalent organic framework thin films. J. Am. Chem. Soc..

[102] Li Y, Wu Q, Guo X, Zhang M, Chen B, Wei G, Li X, Li X, Li S, Ma L (2020). Laminated self-standing covalent organic framework membrane with uniformly distributed subnanopores for ionic and molecular sieving. Nat. Commun..

[103] Kang ZX, Peng YW, Qian YH, Yuan DQ, Addicoat MA, Heine T, Hu ZG, Tee Z, Guo ZG, Zhao D (2016). Mixed matrix membranes (MMMs) comprising exfoliated 2D covalent organic frameworks (COFs) for efficient CO_2_ separation. Chem. Mater..

[104] Shen R, Huang L, Liu R, Shuai Q (2021). Determination of sulfonamides in meat by monolithic covalent organic frameworks based solid phase extraction coupled with high-performance liquid chromatography-mass spectrometric. J. Chromatogr. A.

[105] Karak S, Dey K, Torris A, Halder A, Bera S, Kanheerampockil F, Banerjee R (2019). Inducing disorder in order: hierarchically porous covalent organic framework nanostructures for rapid removal of persistent organic pollutants. J. Am. Chem. Soc..

[106] Liu R, Yan Q, Tang Y, Liu R, Huang L, Shuai Q (2022). NaCl template-assisted synthesis of self-floating COFs foams for the efficient removal of sulfamerazine. J. Hazard. Mater..

[107] Zhang S, Wu X, Ma C, Li Y, You J (2020). Cationic surfactant modified 3D COF and its application in the adsorption of UV filters and alkylphenols from food packaging material migrants. J. Agric. Food Chem..

[108] Cui WR, Zhang CR, Jiang W, Liang RP, Qiu JD (2019). Covalent organic framework nanosheets for fluorescence sensing via metal coordination. ACS Appl. Nano Mater..

[109] Cui WR, Zhang CR, Jing W, Liang RP, Wen SH, Peng D, Qiu JD (2019). Covalent organic framework nanosheet-based ultrasensitive and selective colorimetric sensor for trace Hg^2+^ detection. ACS Sustain. Chem. Eng..

[110] Manna A, Maharana AK, Rambabu G, Nayak S, Basu S, Das S (2021). Dithia-crown-ether integrated self-exfoliated polymeric covalent organic nanosheets for selective sensing and removal of mercury. ACS Appl. Polym. Mater..

[111] Kaleeswaran D, Vishnoi P, Murugavel R (2015). [3+3] Imine and β-ketoenamine-tethered fluorescent covalent-organic frameworks for CO2 uptake and nitroaromatic sensing. J. Mater. Chem. C Mater. Opt. Electron. Devices.

[112] Mal A, Mishra RK, Praveen VK, Khayum MA, Banerjee R, Ajayaghosh A (2018). Supramolecular reassembly of self-exfoliated ionic covalent organic nanosheets for label-free detection of double-stranded DNA. Angew. Chem. Int. Ed..

[113] Wang P, Zhou F, Zhang C, Yin SY, Teng L, Chen L, Hu XX, Liu HW, Yin X, Zhang XB (2018). Ultrathin two-dimensional covalent organic framework nanoprobe for interference-resistant two-photon fluorescence bioimaging. Chem. Sci. (Cambridge).

[114] Wang JM, Lian X, Yan B (2019). Eu^3+^-functionalized covalent organic framework hybrid material as a sensitive turn-on fluorescent switch for levofloxacin monitoring in serum and urine. Inorg. Chem..

[115] Wang J, Yan B (2019). Improving covalent organic frameworks fluorescence by triethylamine pinpoint surgery as selective biomarker sensor for diabetes mellitus diagnosis. Anal. Chem..

[116] Zhang Y, Shen X, Feng X, Xia H, Mu Y, Liu X (2016). Covalent organic frameworks as pH responsive signaling scaffolds. Chem. Commun. (Cambridge).

[117] Yin HQ, Yin F, Yin XB (2019). Strong dual emission in covalent organic frameworks induced by ESIPT. Chem. Sci. (Cambridge).

[118] Mulzer CR, Shen L, Bisbey RP, McKone JR, Zhang N, Abruña HD, Dichtel WR (2016). Superior charge storage and power density of a conducting polymer-modified covalent organic framework. ACS Cent. Sci..

[119] Wu Y, Yan D, Zhang Z, Matsushita MM, Awaga K (2019). Electron highways into nanochannels of covalent organic frameworks for high electrical conductivity and energy storage. ACS Appl. Mater. Interfaces..

[120] Xu Y, Lin Z, Huang X, Wang Y, Huang Y, Duan X (2013). Functionalized graphene hydrogel-based high-performance supercapacitors. Adv. Mater..

[121] Chandra S, Chowdhury DR, Addicoat M, Heine T, Paul A, Banerjee R (2017). Molecular level control of the capacitance of two-dimensional covalent organic frameworks: role of hydrogen bonding in energy storage materials. Chem. Mater..

[122] Halder A, Ghosh M, Khayum MA, Bera S, Addicoat M, Sasmal HS, Karak S, Kurungot S, Banerjee R (2018). Interlayer hydrogen-bonded covalent organic frameworks as high-performance supercapacitors. J. Am. Chem. Soc..

[123] He Y, Chen W, Li X, Zhang Z, Fu J, Zhao C, Xie E (2013). Freestanding three-dimensional graphene/MnO_2_ composite networks as ultralight and flexible supercapacitor electrodes. ACS Nano.

[124] Niu W, Liu J, Mai Y, Müllen K, Feng X (2019). Synthetic engineering of graphene nanoribbons with excellent liquid-phase processability. Trends Chem..

[125] Niu W, Ma J, Soltani P, Zheng W, Liu F, Popov AA, Weigand JJ, Komber H, Poliani E, Casiraghi C, Droste J, Hansen MR, Osella S, Beljonne D, Bonn M, Wang HI, Feng X, Liu J, Mai Y (2020). A curved graphene nanoribbon with multi-edge structure and high intrinsic charge carrier mobility. J. Am. Chem. Soc..

[126] Khayum MA, Vijayakumar V, Karak S, Kandambeth S, Bhadra M, Suresh K, Acharambath N, Kurungot S, Banerjee R (2018). Convergent covalent organic framework thin sheets as flexible supercapacitor electrodes. ACS Appl. Mater. Interfaces..

[127] Sharma RK, Yadav P, Yadav M, Gupta R, Rana P, Srivastava A, Zbořil R, Varma RS, Antonietti M, Gawande MB (2020). Recent development of covalent organic frameworks (COFs): synthesis and catalytic (organic-electro-photo) applications. Mater. Horiz..

[128] Pachfule P, Acharjya A, Roeser J, Langenhahn T, Schwarze M, Schomäcker R, Thomas A, Schmidt J (2018). Diacetylene functionalized covalent organic framework (COF) for photocatalytic hydrogen generation. J. Am. Chem. Soc..

[129] Sheng JL, Dong H, Meng XB, Tang HL, Yao YH, Liu DQ, Bai LL, Zhang FM, Wei JZ, Sun XJ (2019). Effect of different functional groups on photocatalytic hydrogen evolution in covalent-organic frameworks. ChemCatChem.

[130] Wang X, Chen L, Chong SY, Little MA, Wu Y, Zhu WH, Clowes R, Yan Y, Zwijnenburg MA, Sprick RS, Cooper AI (2018). Sulfone-containing covalent organic frameworks for photocatalytic hydrogen evolution from water. Nat. Chem..

[131] Thote J, Aiyappa HB, Deshpande A, Díaz Díaz D, Kurungot S, Banerjee R (2014). A covalent organic framework-cadmium sulfide hybrid as a prototype photocatalyst for visible-light-driven hydrogen production. Chemistry (Weinheim an der Bergstrasse, Germany).

[132] Zhang FM, Sheng JL, Yang ZD, Sun XJ, Tang HL, Lu M, Dong H, Shen FC, Liu J, Lan YQ (2018). Rational design MOF/COF hybrid materials for photocatalytic H_2_ evolution in the presence of sacrificial electron donors. Angew. Chem. Commun..

[133] Luo ML, Yang Q, Liu KW, Cao HM, Yan HJ (2019). Boosting photocatalytic H_2_ evolution on g-C_3_N_4_ via covalent organic frameworks (COFs) modifying. Chem. Commun..

[134] Ming J, Liu A, Zhao J, Zhang P, Huang H, Lin H, Xu Z, Zhang X, Wang X, Hofkens J, Roeffaers MBJ, Long J (2019). Hot **π**-electron tunneling of metal insulator-COF nanostructures for efficient hydrogen production. Angew. Chem. Commun..

[135] Zhong W, Sa R, Li L, He Y, Li L, Bi J, Zhuang Z, Yu Y, Zou Z (2019). A covalent organic framework bearing single Ni sites as a synergistic photocatalyst for selective photoreduction of CO_2_ to CO. J. Am. Chem. Soc..

[136] Liu ZL, Huang YQ, Chang SQ, Zhu XL, Fu YH, Ma R, Lu XQ, Zhang FM, Zhu WD, Fan MH (2021). Highly dispersed Ru nanoparticles on a bipyridinelinked covalent organic framework for efficient photocatalytic CO_2_ reduction. Sustain. Energy Fuels.

[137] Guo K, Zhu XL, Peng LL, Fu YH, Ma R, Lu XQ, Zhang FM, Zhu WD, Fan MH (2021). Boosting photocatalytic CO_2_ reduction over a covalent organic framework decorated with ruthenium nanoparticles. Chem. Eng. J..

[138] Lv H, Zhao X, Niu H, He S, Tang Z, Wu F, Giesy JP (2019). Ball milling synthesis of covalent organic framework as a highly active photocatalyst for degradation of organic contaminants. J. Hazard. Mater..

[139] Cao Y, Liu W, Qian J, Cao T, Wang J, Qin W (2019). Porous organic polymers containing sulfur skeleton for visible light degradation of organic dyes. Chem. Asian J..

[140] Patra BC, Khilari S, Manna RN, Mondal S, Pradhan D, Pradhan A, Bhaumik A (2017). A metal-free covalent organic polymer for electrocatalytic hydrogen evolution. ACS Catal..

[141] Zhao X, Pachfule P, Thomas A (2021). Covalent organic frameworks (COFs) for electrochemical applications. Chem. Soc. Rev..

[142] Aiyappa HB, Thote J, Shinde DB, Banerjee R, Kurungot S (2016). Cobalt-modified covalent organic framework as a robust water oxidation electrocatalyst. Chem. Mater..

[143] Gao Z, Yu ZW, Huang YX, He XQ, Su XM, Xiao LH, Yu Y, Huang XH, Luo F (2020). Flexible and robust bimetallic covalent organic frameworks for the reversible switching of electrocatalytic oxygen evolution activity. J. Mater. Chem. A Mater. Energy Sustain..

[144] Zhao X, Pachfule P, Li S, Langenhahn T, Ye M, Schlesiger C, Praetz S, Schmidt J, Thomas A (2019). Macro/microporous covalent organic frameworks for efficient electrocatalysis. J. Am. Chem. Soc..

[145] Zhao X, Pachfule P, Li S, Langenhahn T, Ye M, Tian G, Schmidt J, Thomas A (2019). Silica-templated covalent organic framework-derived Fe-N-doped mesoporous carbon as oxygen reduction electrocatalyst. Chem. Mater..

[146] Gu S, Wu S, Cao L, Li M, Qin N, Zhu J, Wang Z, Li Y, Li Z, Chen J, Lu Z (2019). Tunable redox chemistry and stability of radical intermediates in 2D covalent organic frameworks for high performance sodium ion batteries. J. Am. Chem. Soc..

[147] Chandra S, Kundu T, Kandambeth S, Babarao R, Marathe Y, Kunjir SM, Banerjee R (2014). Phosphoric acid loaded azo (–N═N–) based covalent organic framework for proton conduction. J. Am. Chem. Soc..

[148] Chandra S, Kundu T, Dey K, Addicoat M, Heine T, Banerjee R (2016). Interplaying intrinsic and extrinsic proton conductivities in covalent organic frameworks. Chem. Mater..

